# Determination of Mechanical Properties of Single and Double-Layer Intraply Hybrid Composites Manufactured by Hand Lay-Up Method

**DOI:** 10.3390/polym18020188

**Published:** 2026-01-09

**Authors:** Mohsen Shams, Ferit Cakir

**Affiliations:** Department of Civil Engineering, Gebze Technical University, 41400 Kocaeli, Türkiye; cakirf@gtu.edu.tr

**Keywords:** intraply hybrid composites (IRC), fiber-reinforced polymers (FRP), hand lay-up, mechanical properties, microstructural characterization, SEM analysis, structural performance

## Abstract

This study experimentally evaluates the mechanical and microstructural performance of single- and double-layer intraply hybrid composite (IRC) laminates produced using the hand lay-up method, focusing on Glass–Aramid (GA), Aramid–Carbon (AC), and Carbon–Glass (CG) configurations. Tensile, flexural, compressive, and density tests were conducted in accordance with relevant ASTM standards to assess the influence of hybrid type and layer number under field-representative manufacturing conditions. Microstructural investigations were performed using optical microscopy and scanning electron microscopy (SEM) to identify fabrication-induced imperfections and their relationship to mechanical behavior. The results demonstrate that increasing the laminate configuration from single to double layer significantly enhances mechanical performance across all hybrid types. Double-layer AC laminates exhibited the highest tensile strength (330.4 MPa) and Young’s modulus (11.93 GPa), corresponding to improvements of approximately 85% and 59%, respectively, compared to single-layer counterparts. In flexural loading, the highest strength was observed in double-layer CG laminates (97.14 MPa), while compressive strength was maximized in double-layer AC laminates (34.01 MPa), indicating improved stability and resistance to compression-driven failure. Statistical analysis confirmed that layer number is the dominant parameter governing mechanical response, exceeding the influence of hybrid configuration alone. Microstructural observations revealed fiber misorientation, incomplete resin impregnation, and localized voids inherent to manual fabrication. However, these imperfections were consistently distributed across all specimens and did not obscure comparative mechanical trends. Coefficients of variation generally remained below 10%, indicating acceptable repeatability despite non-ideal manufacturing conditions.

## 1. Introduction

Fiber-reinforced polymer (FRP) composites have become an important material group in structural engineering due to their low weight, high strength-to-weight ratio, and durability under environmental exposure. In strengthening applications, FRP systems are widely used for the rehabilitation of reinforced concrete elements and other structural components as a practical alternative to steel-based solutions [1]. Among these systems, glass (GFRP), carbon (CFRP), and aramid (AFRP) laminates are commonly preferred because each fiber type provides distinct mechanical advantages: glass fibers offer economical and corrosion-resistant performance, carbon fibers provide high stiffness and tensile capacity, and aramid fibers contribute toughness and impact resistance [1]. Since no individual fiber type satisfies all mechanical demands, hybrid composite laminates have been increasingly adopted to combine multiple desirable properties within a single system [2,3]. Recently, hybrid composites, characterized by the incorporation of different fiber types within the same fabric architecture, have garnered significant attention for their adjustable mechanical performance and superior damage-tolerance potential. Hybrid composites are generally produced in two forms: interply hybrid composites (INPs)—in which fiber types are placed in separate layers—and intraply hybrid composites (IRPs), where fibers are mixed within the same layer [3]. Especially, IRCs have recently emerged as promising alternatives within the broader family of FRP materials. Unlike traditional unidirectional or fabrics composed of a single fiber type, IRCs combine two or more fiber species within the same woven architecture. By strategically integrating fibers such as carbon, glass, and aramid, hybridization offers the possibility of tailoring mechanical properties to meet specific structural demands. For instance, carbon fibers provide high stiffness, glass fibers enhance cost-efficiency and impact resistance, and aramid fibers introduce superior toughness and energy absorption capacity. The interaction of these fibers within a single fabric can yield balanced performance attributes not attainable by monolithic composite systems. Previous studies have shown that intraply hybridization can promote more uniform stress transfer and improve crack-bridging capacity, leading to enhanced mechanical performance compared with interply systems, particularly in carbon–aramid and carbon–glass combinations [4]. Cakir et al. [5] investigated the effectiveness of Intraply Hybrid Composites (IRCs) for enhancing the shear performance of reinforced concrete (RC) beams without transverse reinforcement. Three IRC configurations—Aramid–Carbon, Glass–Aramid, and Carbon–Glass—were applied using U-shaped wrapping and tested under four-point bending. The results demonstrated that all IRC types significantly improved shear capacity and altered the failure mechanisms compared to the unstrengthened beam. Digital image correlation and conventional instrumentation confirmed the enhanced structural response, indicating that IRCs provide a promising alternative to traditional strengthening systems in RC shear applications. Similarly, Cakir et al. [6] conducted a study on the effectiveness of non-hybrid composites (NHCs) and intraply hybrid composites (IRCs) in enhancing the mechanical performance of concrete. Using various specimen geometries, the research evaluates how different composite types—Carbon, Glass, Aramid, and their hybrid combinations—affect key mechanical properties such as compressive and flexural strength, modulus of elasticity, and Poisson’s ratio. The results showed that both NHCs and IRCs lead to significant improvements in concrete behavior, highlighting their potential as efficient reinforcement materials for structural applications. Altaee and Mostafa [7] evaluated the mechanical performance of interply and intraply hybrid jute–glass fiber/epoxy composites in comparison with pure jute and pure glass laminates. Various hybrid configurations were fabricated and tested under tensile, flexural, and impact loading. The findings showed that increasing the glass content enhanced the tensile behavior of interply hybrids, while intraply hybrids exhibited distinct strength–stiffness trade-offs depending on yarn arrangement. Junaedi and Sebaey [8] examined the mechanical behavior of carbon–aramid hybrid epoxy laminates containing open holes and single-edge notches. The hybrid configuration, in which aramid fibers were sandwiched between carbon layers, was evaluated against pure CFRP and AFRP laminates under tensile and flexural loading. The results showed that the hybrid laminate exhibited lower notch sensitivity than CFRP and displayed a mixed failure pattern combining AFRP fiber breakage with CFRP outer-layer delamination. The hybrid also achieved the highest flexural modulus among all laminates and demonstrated fracture toughness greater than CFRP but lower than AFRP, confirming the benefits of combining carbon’s strength with aramid’s toughness. Agarwal et al. [9] studied basalt/epoxy composites modified with carbon–Kevlar intraply surface layers and found that the hybrid configuration significantly improved mechanical strength, impact resistance, damping, and sound insulation compared with pure basalt laminates, supported by SEM evidence of enhanced toughness mechanisms. Islam et al. [10] investigated the fatigue performance of flax–carbon hybrid composites and showed that intraply hybridization achieved a dramatically longer fatigue life than interply layouts, demonstrating that properly designed natural–synthetic hybrids can rival fully synthetic composites while maintaining high bio-based content. Huang et al. [11] studied demonstrates that pseudo-ductility in 3D printed composite laminates can be achieved by hybridizing low-elongation and high-elongation fibers, where low-strain material (LSM) layers promote controlled fragmentation and delay brittle failure. The pseudo-yield response is shown to depend on LSM thickness, LE-to-HE material ratio, and the encapsulating effect of high-elongation fibers, highlighting the potential of additive manufacturing for tailoring metal-like mechanical behavior in composites. Zhu et al. [12] investigated low-velocity impact damage in eco-friendly flax/basalt intraply hybrid PP composites using infrared thermography and THz-TDS, demonstrating that multi-source data fusion significantly improves damage detection, resin distribution assessment, and overall impact resistance evaluation compared to single-sensor approaches. Özbek et al. [13] examine the influence of hydrothermal aging on the crashworthiness of intraply glass/basalt fiber-reinforced composite pipes manufactured by wet filament winding. Results show that hybridisation reduces water uptake compared to non-hybrid basalt pipes and promotes more stable, progressive crushing behavior under quasi-static axial compression, although aging and increased winding angle lead to reduced energy absorption and load-carrying capacity. Jeyaguru et al. [14] conducted the thermal and thermomechanical performance of intra-ply Kevlar/hemp fiber–reinforced epoxy composites with different weaving patterns, showing that plain and basket weaves provide the highest thermal stability, while twill weave exhibits superior dynamic mechanical properties. Overall, all hybrid configurations demonstrate enhanced thermal performance, indicating their suitability for thermal insulation, fire-resistant, and automotive applications. Hashim et al. [15] examined the effect of fiber orientation on the tensile and low-cycle fatigue behavior of intraply carbon/Kevlar hybrid composites, showing that carbon-aligned specimens provide the highest strength and stiffness, while off-axis loading yields increased ductility due to fiber rotation. Fatigue results indicate slower degradation and enhanced durability in the Kevlar loading direction, attributed to fiber stiffening and distinct fatigue damage phases. Brands et al. [16] compared oscillatory and transient torsion bar techniques for evaluating the in-plane shear behavior of unidirectional thermoplastic composites in the melt state, showing that while small-strain oscillatory tests align with transient results at low deformations, transient torsion induces significant anisotropic and through-thickness shear. The findings highlight limitations of transverse isotropy assumptions and question the reliability of transient torsion rheometry despite improvements achieved with a nonlinear anisotropic viscoelastic model. Dong [17] employed an FEA-based approach to investigate the flexural behavior of carbon/glass fiber hybrid composites, demonstrating that delamination governs flexural failure and is strongly influenced by stacking sequence. Predicted flexural strengths based on delamination criteria show close agreement with experimental results, highlighting the critical role of hybrid layup design in composite performance.

Most experimental research on hybrid laminates has been conducted using controlled manufacturing techniques such as vacuum-assisted resin transfer molding (VARTM) or autoclave processing [18,19,20,21,22,23,24,25]. While these methods minimize voids and ensure stable fiber wetting, they do not fully represent field conditions. In the practical applications, the hand lay-up method remains the most widely used because of its simplicity, low cost, and flexibility, even though it can introduce non-uniform fiber wetting, variable resin content, and curing inconsistencies that influence final laminate behavior. Although intraply hybrid systems are gaining interest, systematic comparisons between single-layer and double-layer intraply laminates produced by hand lay-up are still limited.

Therefore, the present study investigates the mechanical performance of single- and double-layer intraply hybrid laminates manufactured using the hand lay-up technique. Laminates incorporating aramid, carbon, and glass fibers were produced in both monolithic and hybrid forms, and their mechanical behavior was evaluated through tensile, flexural (three-point bending), compressive, and density measurements. The aim is to characterize the intrinsic response of these laminates under practical fabrication conditions and to provide a performance baseline for comparison with industrial manufacturing methods such as VARTM.

The scientific novelty of this study resides in its explicit focus on intraply hybrid composite laminates manufactured via the hand lay-up method under field-representative conditions, combined with a systematic comparison between single-layer and double-layer configurations. While intraply hybrid composites have been widely investigated, most existing studies rely on controlled manufacturing techniques such as VARTM or autoclave curing. In contrast, the present work establishes a mechanical performance baseline for IRC laminates fabricated using hand lay-up, which remains the dominant technique in on-site structural strengthening applications.

**Figure 1:**
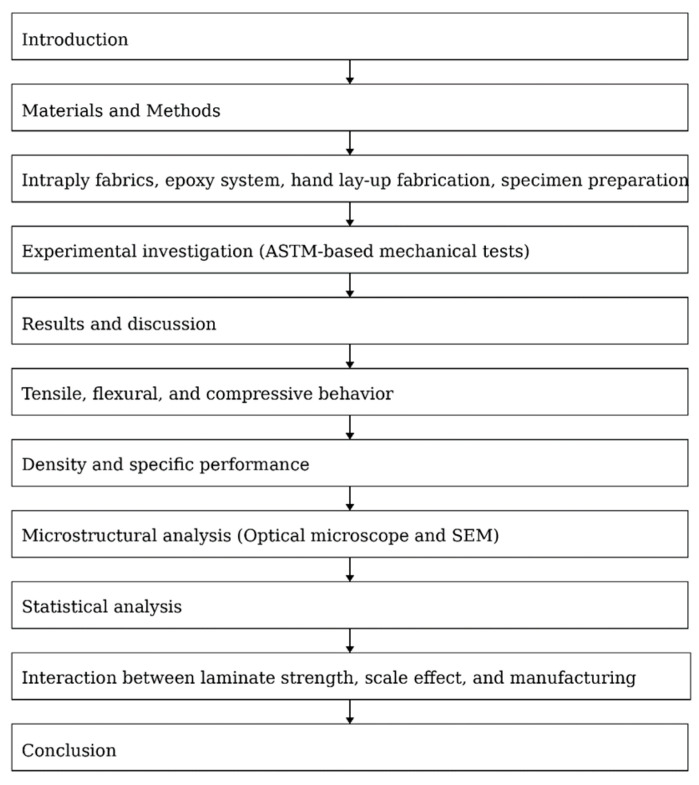
Flowchart of the Study.

Furthermore, the study integrates statistical significance analysis, density-normalized performance metrics, and microstructural observations to link hybrid architecture, layer scaling, and manufacturing-induced imperfections. This combined approach bridges the gap between idealized laboratory studies and practical civil engineering applications (Figure 1).

## 2. Materials and Methods

### 2.1. Intraply Hybrid Fabrics

**Figure 2:**
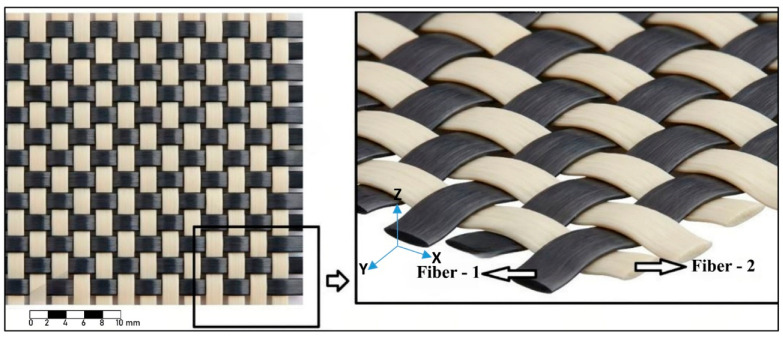
Intraply Hybrid Fabrics [1].

Intraply hybrid fabrics are engineered woven textiles in which two distinct fiber types are integrated within the same fabric layer, enabling direct fiber–fiber interaction and controlled distribution of mechanical properties at the yarn scale. Unlike interply hybrid systems—where each fiber type is placed in separate composite layers—intraply hybrid fabrics achieve hybridization directly within the woven architecture, allowing both warp and weft directions to carry mixed fiber constituents (Figure 2).

In the present study, three different intraply hybrid fabrics were utilized. Each fabric was produced by combining carbon, aramid, and glass yarns in predetermined sequences while maintaining identical weave geometry, yarn density, and overall areal weight across all fabric types. The hybrid configurations are summarized as follows:

Glass–Aramid (GA) Hybrid Fabric: This fabric integrates glass yarns, which contribute dimensional stability and moderate stiffness, with aramid yarns that provide high toughness and energy absorption. The resulting textile offers a compliant and damage-tolerant reinforcement architecture suitable for applications requiring distributed deformation capacity.

Aramid–Carbon (AC) Hybrid Fabric: Aramid fibers enhance ductility and crack-arresting capability, while carbon fibers supply high stiffness and tensile strength. Their combined use within a single woven layer creates a balanced hybrid textile that blends strength and toughness without requiring separate plies.

Carbon–Glass (CG) Hybrid Fabric: Carbon yarns deliver high axial stiffness and load-carrying capability, whereas glass yarns enhance strain compatibility and help stabilize the fabric geometry. This pairing produces a cost-efficient hybrid fabric with favorable stiffness-to-weight characteristics.

All hybrid fabrics were prepared with controlled fiber sequencing in both warp and weft directions, ensuring that the only variable among configurations was the fiber type itself. These intraply hybrid textiles served as the reinforcement phase in the composite laminates presented in subsequent sections, where matrix application and laminate fabrication are described separately.

### 2.2. Epoxy System

All composite laminates in this study were produced using Teknobond 300 TIX (Tekno Construction Chemicals, Istanbul, Türkiye), a two-component thixotropic epoxy system designed specifically for structural strengthening and fiber-reinforced composite applications. The resin (Part A) and hardener (Part B) were mixed according to the manufacturer’s recommended ratio to ensure proper stoichiometric balance and full crosslinking during curing.

Teknobond 300 TIX was selected for its favorable rheological behavior, which is particularly advantageous for hand lay-up fabrication. Its thixotropic nature prevents excessive resin flow, enhances wet-out of carbon, aramid, and glass yarns, and assists in maintaining uniform resin distribution throughout the intraply architecture. The system also provides strong adhesion to a wide range of fibers and substrates, contributing to efficient stress transfer between hybrid fiber constituents. The epoxy exhibits stable curing characteristics under ambient laboratory conditions, allowing laminates to be fabricated without the need for elevated-temperature curing cycles. Its working time, gelation behavior, and final hardness are suitable for manual composite manufacturing where gradual resin penetration and controlled laminate consolidation are required. After curing, the matrix provides adequate stiffness, cohesive strength, and interfacial bonding quality to support the mechanical evaluation of hybrid laminate configurations. A summary of the technical properties of the epoxy system is presented in Table 1.

### 2.3. Fabrication of Composite Laminates

Composite laminates in this study were fabricated using the hand lay-up technique, which represents the most widely used field-application method in construction engineering, particularly for externally bonded FRP strengthening systems. The choice of this technique is aligned with the primary aim of the study—to evaluate intraply hybrid fabrics under fabrication conditions that closely resemble real on-site practices rather than highly controlled industrial processes such as VARTM or autoclave curing. In hand lay-up production, the impregnation of fibers, the consolidation of layers, and the curing process are all governed by manual operations, making it essential to document the technique precisely since it strongly influences the final mechanical behavior of hybrid composite laminates [26].

**Figure 3:**
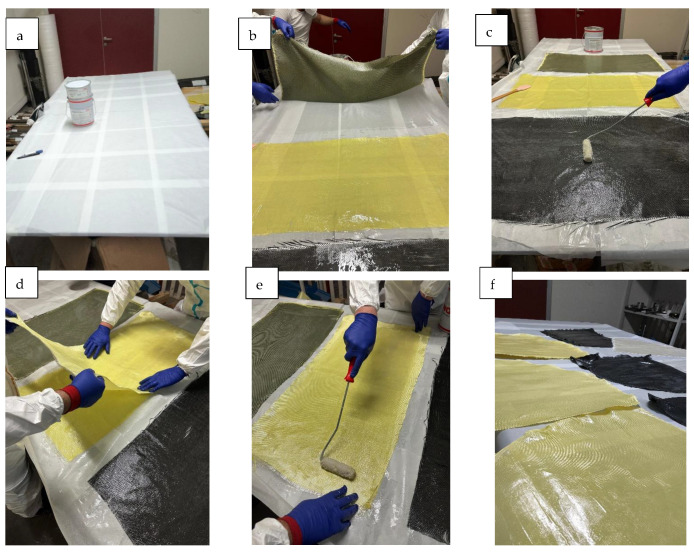
(**a**) Preparation of the workbench and resin system, (**b**) Cutting and alignment of the first fabric ply, (**c**) Manual resin impregnation of the first ply, (**d**) Placement and alignment of the second fabric ply, (**e**) Manual resin impregnation of the second ply, (**f**) Ambient conditioning of the fabricated laminates prior to specimen preparation.

Before the lay-up process, each intraply hybrid fabric (GA, AC, and CG) was cut to the required dimensions, ensuring proper warp alignment to preserve the intended yarn orientation. Specimens were cut such that the primary loading direction (tensile, flexural, and compressive) aligned with the fiber yarn denoted by the first letter in the hybrid designation (AC, GA, and CG). Accordingly, in AC laminates the loading direction was aligned with the carbon yarns, in GA laminates with the glass yarns, and in CG laminates with the carbon yarns. This convention was adopted to ensure consistency in specimen orientation and to enable direct comparison of mechanical responses governed by the fiber system aligned with the applied load. A thin, uniform layer of epoxy resin was applied to the mold surface to promote adhesion and prevent the formation of dry interfaces. Subsequently, the fabric was placed onto the mold, and the Teknobond 300 TIX epoxy system was manually distributed across the textile using rollers and spreaders (Figure 3). Owing to its thixotropic nature, the resin allowed controlled penetration into the mixed fiber bundles without excessive flow, a feature that is particularly important for intraply hybrid fabrics containing both hydrophobic and hydrophilic yarns. The distinct wetting behaviors of carbon, aramid, and glass fibers required a gradual and meticulously controlled impregnation process to ensure uniform saturation. This stage is critical because insufficient wet-out of carbon bundles, resin-repelling behavior of aramid fibers, or excessive absorption by glass fibers can create resin-rich zones or voids, leading to inconsistent mechanical performance. During the hand lay-up process, air pockets were reduced through manual impregnation and repeated rolling/pressing using a silicon spatula, which is a standard practice in field-applied FRP installations. This procedure was applied consistently to all specimens to improve fiber wet-out and to minimize entrapped air as much as reasonably possible under non-vacuum conditions. However, it is well recognized that complete elimination of voids is not achievable using the hand lay-up technique alone, without vacuum-assisted or pressure-controlled consolidation. Accordingly, despite the use of spatula-based compaction, minor voids and resin-rich regions remained present in the laminates.

For double-layer laminates, a second hybrid fabric sheet was placed directly over the partially impregnated first layer, after which resin application continued until complete saturation was achieved. Since no vacuum bagging or mechanical pressing was used, consolidation depended entirely on manual rolling and the inherent viscosity of the resin. This approach intentionally reflects field-level composite production, where external pressure or controlled curing environments are typically unavailable. As a result, natural process variations such as local fiber volume fraction fluctuations, minor surface undulations, microvoids, and small resin pockets were not artificially suppressed; instead, they were recognized as inseparable characteristics of hand lay-up composites that may influence the structural response.

After the lay-up stage, the laminates were left to cure under ambient laboratory conditions without any applied pressure or elevated temperature. The curing process proceeded through initial gelation and subsequent matrix crosslinking, during which the laminates were kept undisturbed to avoid yarn displacement or resin pooling. Once curing was complete, the composite panels were subjected to visual examination to identify common hand lay-up defects including regions of insufficient resin penetration, entrapped air, fiber pull-up at edges, and localized thickness variations. Panels exhibiting excessive or non-uniform defects were excluded to ensure consistency within the mechanical testing program. The resulting laminates thus represent the mechanical potential of intraply hybrid fabrics when produced under realistic, field-representative manufacturing conditions.

### 2.4. Specimen Preparation

**Figure 4:**
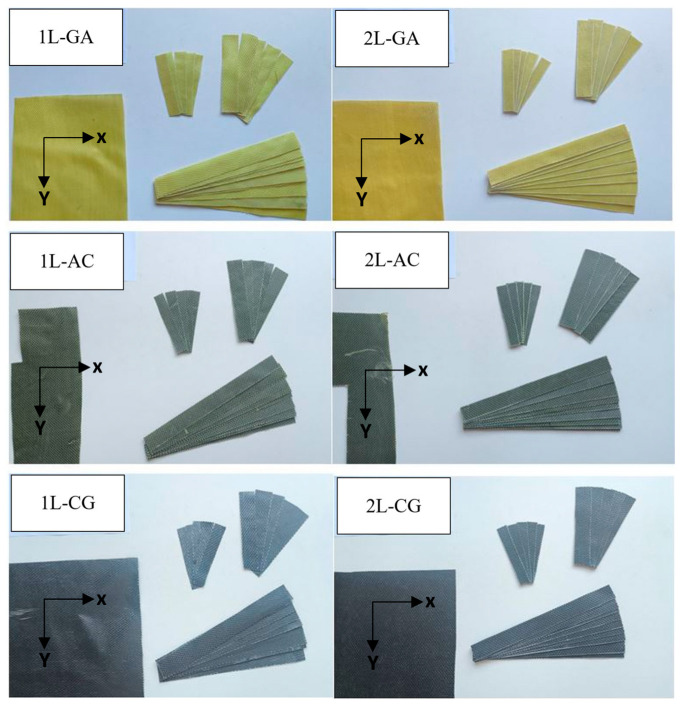
IRCs Test Samples.

Following complete ambient curing of the composite panels, mechanical test specimens were prepared in accordance with the relevant ASTM standards to ensure consistency and comparability across all laminate configurations (Table 2). Prior to specimen extraction, each composite plate was visually inspected and trimmed to remove edge irregularities associated with the hand lay-up process. The plates were then sectioned into individual coupons using a precision cutting device suitable for fiber-reinforced polymer laminates, ensuring smooth cut surfaces and minimizing damage to the fibers and epoxy matrix. During cutting, care was taken to avoid excessive mechanical or thermal loading that could adversely affect the fiber–matrix interface, which is particularly important for hybrid laminates containing carbon, aramid, and glass fibers with different mechanical responses. The cutting procedure resulted in clean and uniform specimen edges without visible delamination or fiber pull-out. After cutting, all specimen edges were manually wet-sanded using progressively finer abrasive papers to remove minor surface imperfections and machining-induced micro-notches that could otherwise act as premature crack initiation sites during mechanical testing. The specimens were then cleaned with isopropyl alcohol to remove surface debris and contaminants prior to testing (Figure 4). To ensure stable moisture conditions and to reduce variability associated with the different hygroscopic characteristics of the constituent fibers, all specimens were conditioned under laboratory ambient conditions for a minimum of 48 h prior to mechanical characterization. This conditioning step is particularly relevant for intraply hybrid laminates, as carbon, aramid, and glass fibers may respond differently to environmental moisture, potentially influencing local strain distribution and elastic behavior. Specimen dimensions, number of layers, and laminate thicknesses were measured and recorded for each configuration prior to testing and are reported in the corresponding experimental sections. Laminate thickness was determined as the average of multiple measurements taken along the gauge length to account for minor thickness variations inherent to hand lay-up fabrication. Through this controlled preparation procedure, the tested specimens reliably represent both the intrinsic characteristics of hand lay-up–manufactured intraply hybrid laminates and the boundary conditions imposed by standardized mechanical test configurations. The careful control of cutting, surface finishing, conditioning, and dimensional verification ensures that the mechanical responses reported in the Results and Discussion section primarily reflect the influence of the hybrid laminate architecture and fabrication method, rather than artefacts associated with specimen preparation.

## 3. Experimental Investigation of IRC Laminates

The mechanical behavior of the IRC laminates was evaluated through a comprehensive experimental program designed to characterize their tensile, flexural, compressive, and density-related properties. All tests were performed under controlled laboratory conditions using standardized procedures to ensure repeatability and to allow reliable comparisons among the three hybrid configurations (GA, AC, and CG). Each test method was selected to capture a different aspect of the structural response of the laminates, ranging from in-plane load-bearing capacity and stiffness to out-of-plane bending resistance, compressive stability, and material-specific density characteristics. For each laminate configuration and each test type (tensile, flexural, compressive, and density), five specimens were tested. The experimental program also included detailed visual documentation, where failure modes, crack initiation zones, fiber–matrix debonding characteristics, and overall fracture patterns were recorded using digital imaging.

In the following subsections, each test category is presented individually. The corresponding test setups, boundary conditions, specimen geometries, measurement principles, and relevant ASTM standards are described in detail. Representative test images captured during loading are also provided to illustrate the deformation mechanisms and failure processes associated with each hybrid configuration. Together, these experimental observations form the basis for the subsequent analysis and discussion of the mechanical performance of the intraply hybrid composite laminates.

### 3.1. Tensile Tests

**Figure 5:**
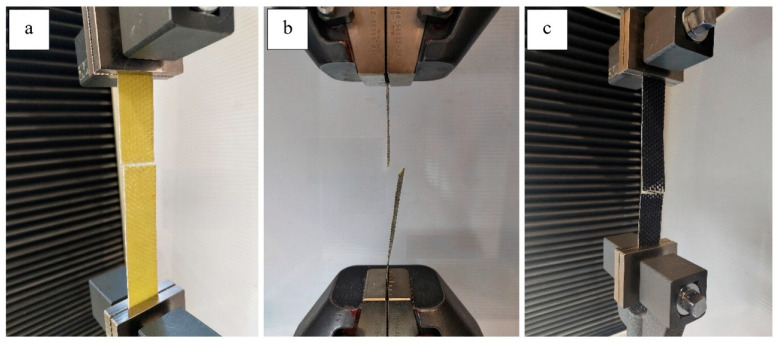
Tensile Test Setup; (**a**) 2L-GA, (**b**) 1L-AC, (**c**) 2L-CG.

**Figure 6:**
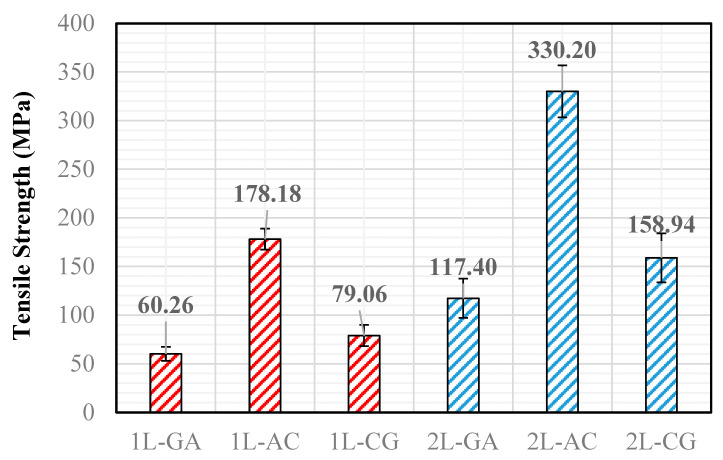
Tensile Strength Values obtained from the Tests.

Tensile behavior of the intraply hybrid composite laminates was evaluated in accordance with ASTM D3039 [27], and the resulting tensile strengths for both single-layer (1L) and double-layer (2L) configurations are presented in Figure 5. The results reveal a strong dependence of tensile performance on both the hybrid fabric type and the number of intraply layers. Among the single-layer laminates, the Aramid–Carbon configuration (1L-AC) achieved the highest tensile strength (178.18 MPa), reflecting the dominant load-carrying contribution of carbon fibers supported by the crack-bridging capability of aramid yarns. The Carbon–Glass laminate (1L-CG) exhibited an intermediate strength level (79.06 MPa), consistent with a balanced but limited synergy between carbon stiffness and the lower tensile efficiency of glass fibers. The Glass–Aramid hybrid (1L-GA) showed the lowest tensile capacity (60.26 MPa), which is expected given the absence of carbon fibers and the inherently lower stiffness of the glass–aramid pairing. A pronounced improvement in tensile strength was observed when transitioning from single-layer to double-layer laminates. The increase was most significant for the Carbon–Glass system, where the tensile strength doubled from 79.06 MPa to 158.94 MPa (≈101% increase). The Glass–Aramid hybrid showed a similarly high improvement, rising from 60.26 MPa to 117.40 MPa (≈95% increase). The Aramid–Carbon laminate exhibited the highest absolute tensile strength among all configurations, increasing from 178.18 MPa to 330.20 MPa (≈85% increase) (Figure 6). These results indicate that multilayer stacking substantially enhances load transfer efficiency, improves structural stability, and reduces the influence of local imperfections typically introduced during hand lay-up fabrication. Overall, the tensile test results demonstrate that the tensile performance hierarchy AC > CG > GA remains consistent in both the 1L and 2L configurations, while the magnitude of improvement with additional layers varies depending on the fiber pairing. AC laminates benefit from the synergistic interaction between high-modulus carbon and tough aramid fibers, whereas the CG and GA systems show greater proportional gains due to the enhanced consolidation and reduced defect sensitivity provided by the second layer. Representative failure photographs presented in the following figures illustrate the corresponding fracture patterns, including fiber rupture, matrix cracking, and local delamination, which further support the mechanical trends observed in the tensile tests.

**Figure 7:**
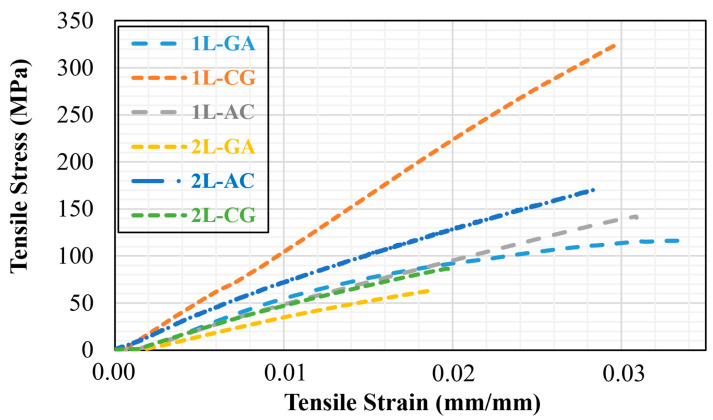
The Stress–Strain Curves obtained from the Tensile Tests.

**Figure 8:**
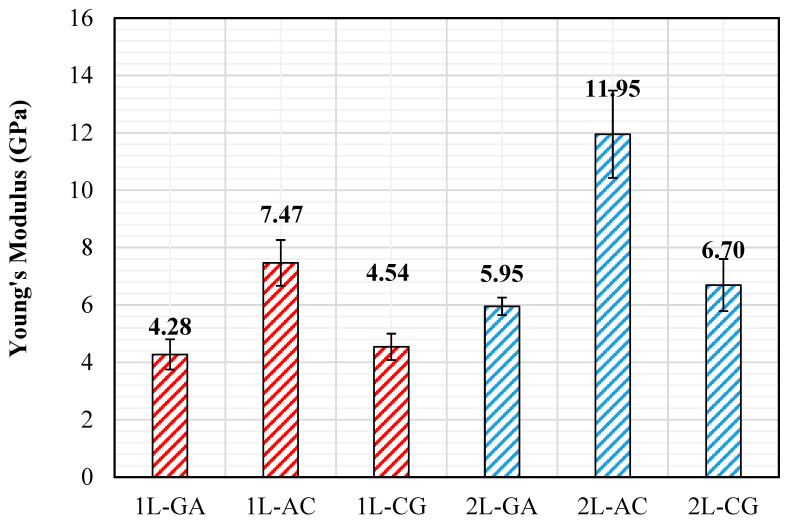
The Young’s Modulus obtained from the Tensile Tests.

The stress–strain curves clearly show that the tensile behavior of the intraply hybrid laminates depends strongly on both the fiber combination and the number of layers. Among the single-layer systems, 1L-CG exhibits the highest initial stiffness, 1L-AC shows a balanced stiffness–ductility response, and 1L-GA remains the most compliant configuration. Adding a second layer enhances performance in all hybrids: the 2L-AC laminate reaches the highest overall stress levels, while 2L-CG and 2L-GA also shift upward, reflecting improved consolidation and load transfer. The increase from 1L to 2L reduces the influence of local imperfections typical of hand lay-up fabrication and results in smoother, more stable stress–strain responses. Overall, AC hybrids provide the best strength, CG hybrids deliver high stiffness, and GA hybrids maintain the greatest strain capacity. The Young’s modulus results confirm the stiffness hierarchy suggested by the stress–strain curves. Among the single-layer laminates, 1L-AC exhibits the highest stiffness (7.47 GPa) due to the dominant contribution of carbon fibers, while 1L-GA (4.28 GPa) and 1L-CG (4.54 GPa) remain significantly more compliant (Figure 7). The addition of a second intraply layer consistently increases stiffness across all hybrids, but the magnitude of improvement varies. The 2L-AC laminate shows the most substantial increase, reaching 11.95 GPa, which reflects enhanced fiber alignment stability and improved load distribution in the multilayer configuration. The CG and GA system salso display stiffness gains (6.70 GPa and 5.95 GPa, respectively), though these increases are more modest, consistent with the lower inherent modulus of glass and aramid fibers. Overall, the modulus results reinforce the conclusion that AC hybrids deliver the highest stiffness, CG hybrids provide moderate stiffness, and GA hybrids remain the most flexible, while the transition from 1L to 2L consistently improves structural rigidity for all configurations (Figure 8).

### 3.2. Flexural Tests

**Figure 9:**
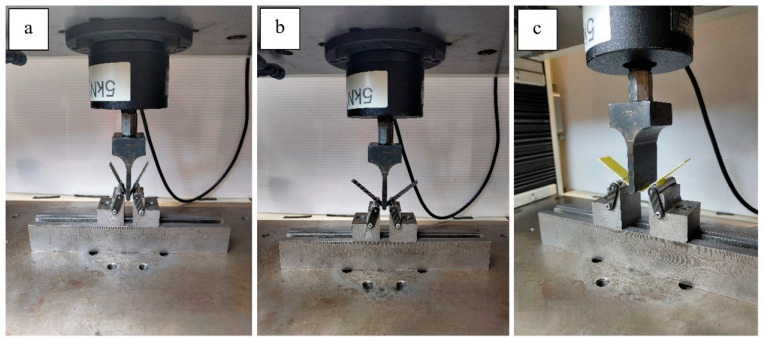
Flexural test setup; (**a**) 1L-AC, (**b**) 1L-CG, (**c**) 2L-GA.

**Figure 10:**
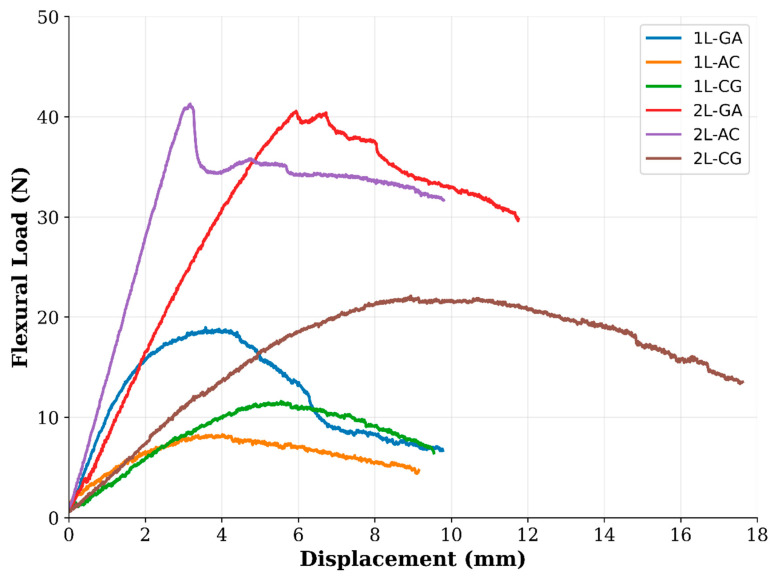
Flexural Loads obtained from the Flexural Tests.

**Figure 11:**
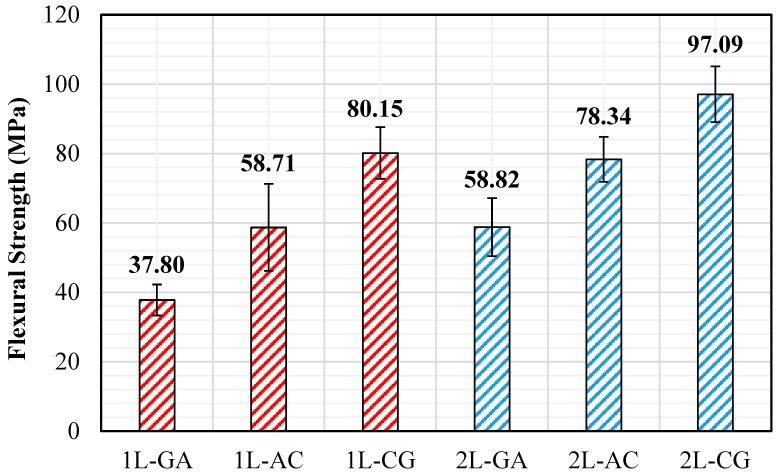
Flexural Strength obtained from the Flexural Tests.

Flexural performance of the intraply hybrid laminates was evaluated using the three-point bending setup prescribed by ASTM D790 [28], and the corresponding flexural strength values and load–displacement responses are presented in Figure 9. The results show that the bending behavior is highly dependent on both the fiber combination within the intraply architecture and the number of laminate layers. Among the single-layer configurations, the 1L-CG laminate exhibits the highest flexural strength (80.15 MPa), reflecting the contribution of carbon fibers to initial bending stiffness while glass fibers help to delay catastrophic tensile-side fracture. The 1L-AC laminate shows moderate flexural capacity (58.71 MPa), whereas 1L-GA displays the lowest value (37.80 MPa) due to the inherently lower bending stiffness associated with glass–aramid interactions. Moving from a single to a double layer significantly enhances bending performance. The 2L-CG laminate reaches the highest flexural strength overall (97.09 MPa), indicating that this hybrid benefits strongly from increased section thickness and improved stress redistribution. The 2L-AC laminate also exhibits a substantial increase (78.34 MPa), whereas 2L-GA shows a more modest improvement (58.82 MPa) but still nearly doubles its 1L counterpart (Figure 10 and Figure 11). These trends confirm that multilayer stacking improves through-thickness stiffness and minimizes the influence of local imperfections and fiber misalignment inherent to hand lay-up fabrication. The flexural load–displacement curves further clarify the mechanical behavior. 1L-GA and 1L-CG display limited load-carrying capacity and early softening, consistent with their lower flexural strength values. 1L-AC, despite moderate flexural strength, shows a rapid load drop after peak, indicating brittle tensile-side fiber rupture. In contrast, the double-layer hybrids exhibit markedly different behavior. 2L-AC and 2L-GA curves show higher initial slopes and extended post-peak regions, demonstrating enhanced bending rigidity and improved energy absorption. The 2L-CG laminate displays the longest displacement capacity and the most gradual load decay, suggesting a more stable bending response enabled by mixed stiffness pathways within the intraply architecture. Overall, the flexural test results indicate that the bending performance hierarchy follows CG > AC > GA for both single- and double-layer laminates, while the relative improvement from 1L to 2L is most pronounced in the CG system. The observed failure modes—matrix cracking, compression-side fiber buckling, tensile-side rupture, and local delamination—correspond well with the load–displacement signatures and confirm that flexural behavior in intraply hybrids is governed by the synergy among carbon, glass, and aramid fibers within the woven textile layer.

### 3.3. Compressive Tests

**Figure 12:**
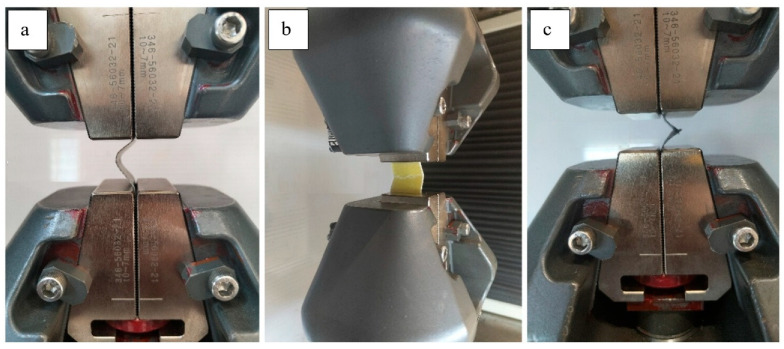
Compression tests; (**a**) 1L-AC, (**b**) 2L-GA, (**c**) 1L-CG.

Compressive behavior of the intraply hybrid laminates was evaluated using the ASTM D3410 [29] shear-loading compression fixture, and the resulting load–displacement responses and compressive strength values are presented in Figure 12. The experimental results reveal a strong influence of both laminate configuration and fiber architecture on compressive stability, peak load capacity, and post-buckling behavior.

**Figure 13:**
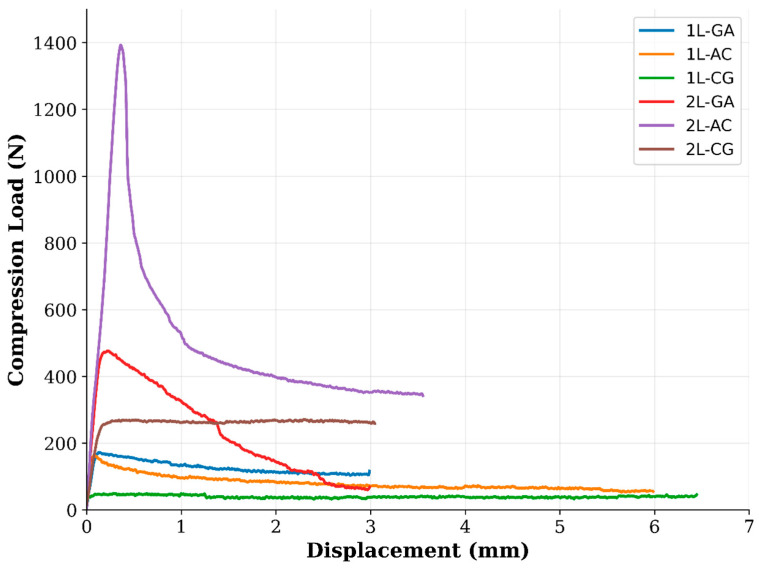
Compressive Loads obtained from the Compressive Tests.

**Figure 14:**
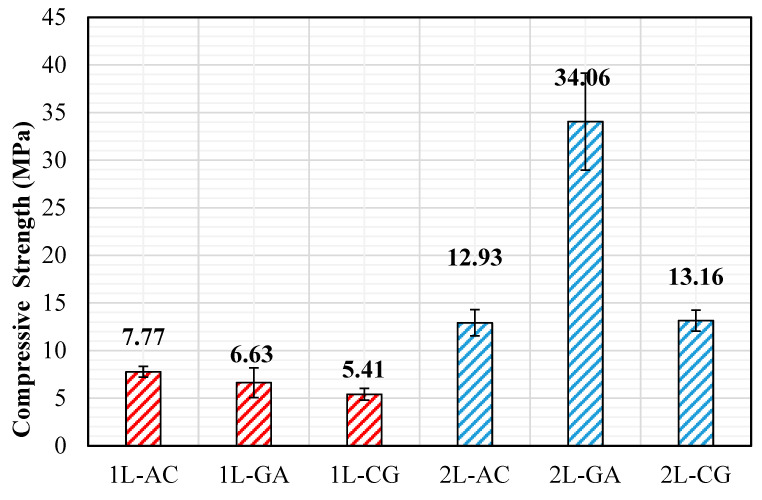
Compressive Strength obtained from the Compressive Tests.

The load–displacement curves show clear distinctions among the hybrid systems. The 2L-AC laminate exhibits the highest peak compression load, reaching values well above 1300 N, followed by a gradual load drop indicative of progressive kink-band formation rather than sudden catastrophic collapse. This behavior reflects the combined stabilizing contribution of carbon fibers—which resist axial deformation until microbuckling initiates—and aramid fibers, which enhance resistance to shear-dominated instability. The 2L-CG laminate demonstrates a lower peak load (≈250–300 N) but maintains a more extended plateau region, suggesting that glass fibers help distribute compressive stresses and delay complete structural degradation. The 2L-GA system displays a moderate peak (≈450–500 N), after which load decreases steadily, indicating a relatively ductile response governed by glass–aramid shear deformation. In contrast, all single-layer (1L) laminates exhibit significantly lower compressive load capacities and sharper post-peak drops. Among them, 1L-GA shows the highest peak (≈250–300 N), whereas 1L-AC and 1L-CG remain considerably lower, consistent with the limited through-thickness stability provided by a single hybrid layer. The abrupt softening observed in 1L-AC and 1L-CG suggests that compressive microbuckling initiates earlier when laminate thickness is small, and local imperfections introduced during hand lay-up fabrication become more critical. The compressive strength values reinforce these trends. While all single-layer hybrids exhibit modest compressive strengths (7.77 MPa for 1L-GA, 6.63 MPa for 1L-AC, 5.41 MPa for 1L-CG), the addition of a second intraply layer produces substantial improvements. The 2L-AC laminate reaches 34.06 MPa, representing a more than five-fold increase relative to its 1L counterpart. The 2L-CG and 2L-GA laminates also show significant enhancements (13.16 MPa and 12.93 MPa, respectively), reflecting improved structural stability and reduced susceptibility to premature fiber microbuckling (Figure 13 and Figure 14).

In summary, the compressive test results demonstrate that multilayer stacking is critical for achieving stable compressive behavior in intraply hybrid laminates. The AC configuration provides the highest compressive strength and stiffness due to the synergistic interaction of carbon and aramid fibers, while the CG and GA hybrids exhibit more moderate improvements. The combination of load–displacement responses and strength data confirms that compressive performance is governed by a balance between axial stiffness, shear resistance, and the ability of the hybrid fabric architecture to suppress local instability mechanisms under increasing compressive strain.

### 3.4. Density Measurements

Density measurements were conducted using the water-immersion method specified in ASTM D792 [30], allowing accurate determination of the mass-to-volume ratio of the hybrid laminates. Each specimen was weighed in air and subsequently while submerged in water, and the density was calculated by incorporating the buoyancy correction. These measurements provide insight into the fiber–matrix distribution within the laminate and are particularly relevant for hand lay-up composites, where resin-rich regions and small voids may cause variations in local density.

Among the single-layer laminates, 1L-CG exhibits the highest density (1.43 g/cm^3^), reflecting the combined contribution of carbon and glass fibers, both of which possess higher intrinsic densities compared to aramid. The 1L-AG and 1L-AC laminates show similar but lower densities (1.30 g/cm^3^ and 1.27 g/cm^3^, respectively), consistent with the presence of aramid fibers, which reduce the overall mass-to-volume ratio due to their relatively low specific gravity. This pattern aligns well with the mechanical results: hybrids containing carbon and glass fibers tend to exhibit higher stiffness and strength, which is compatible with their higher measured densities.

**Figure 15:**
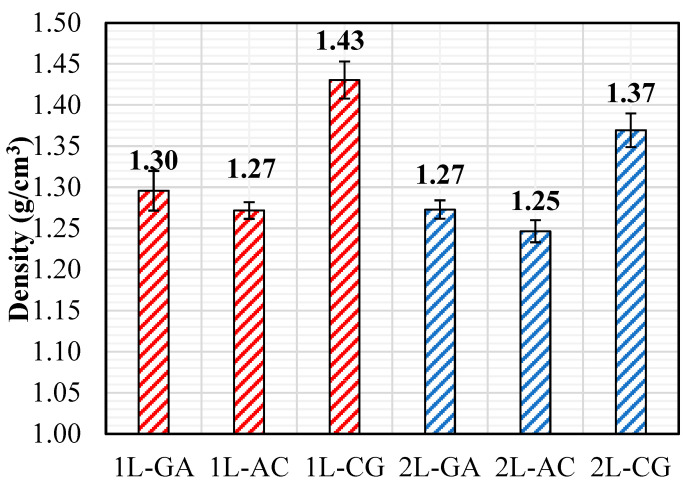
Density Values obtained from the Density Tests.

A similar trend is observed in the double-layer systems. The 2L-CG laminate again exhibits the highest density (1.37 g/cm^3^), followed by 2L-AG (1.27 g/cm^3^) and 2L-AC (1.25 g/cm^3^). Notably, the density increase from 1L to 2L is more pronounced for the CG hybrid, suggesting improved laminate consolidation and reduced void content when two carbon–glass layers are combined (Figure 15). In contrast, the AC and AG systems show relatively minor changes in density with increased thickness, which may reflect the higher resin absorption and slightly greater void tendency associated with aramid-containing fabrics during manual impregnation. Overall, the density measurements correlate well with the mechanical behavior of the laminates. Hybrids incorporating heavier, stiffer fibers (carbon and glass) achieve higher density and correspondingly higher tensile and flexural performance, whereas aramid-containing systems maintain lower density and enhanced ductility. These findings reinforce the link between fiber architecture, laminate consolidation, and the macroscopic mechanical properties of intraply hybrid composites produced via hand lay-up.

## 4. Results and Discussion

The comprehensive mechanical characterization performed in this study demonstrates that intraply hybridization, when combined with the inherent variability of the hand lay-up fabrication method, results in distinct, tunable, and reproducible mechanical responses across tensile, flexural, compressive, and density-normalized (specific) performance metrics. Rather than aiming to achieve idealized material properties, the experimental results provide a realistic representation of how intraply hybrid composite laminates behave under field-representative manufacturing conditions, which is particularly relevant for structural strengthening applications in civil engineering.

The three investigated hybrid configurations (GA, AC, and CG) exhibit clearly differentiated mechanical profiles, confirming that hybrid fabric design at the yarn scale governs stress redistribution and damage evolution under different loading modes. Although detailed fracture mechanics modeling was beyond the scope of this work, macroscopic failure observations consistently indicate that fiber stiffness contrast, intralayer interaction, and layer stacking collectively influence load transfer efficiency and failure progression.

### 4.1. Tensile Behavior

**Figure 16:**
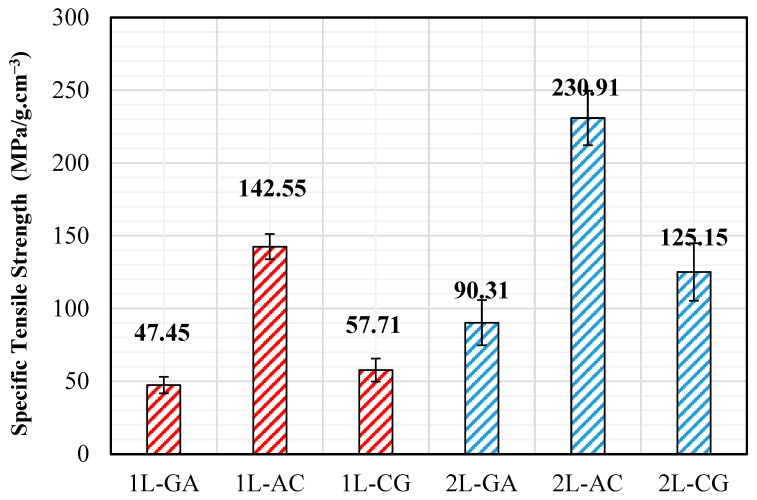
Specific Tensile Strength Values.

Tensile test results reveal a consistent hierarchy governed primarily by fiber stiffness contrast and intraply interaction. Among the tested systems, the AC hybrid exhibits the highest tensile strength and modulus in both single- and double-layer configurations. This behavior can be attributed to the dominant load-carrying role of carbon fibers, while aramid fibers contribute to delayed crack opening and improved strain compatibility through intralayer interaction. The CG system benefits from carbon fiber stiffness; however, its tensile response remains comparatively more brittle, reflecting the limited strain capacity and reduced load-sharing capability of glass fibers within the intraply architecture. In contrast, the GA system demonstrates the lowest tensile stiffness but the highest deformation capacity, indicating a more compliant and damage-tolerant response dominated by aramid–glass interaction. Layer stacking significantly amplifies tensile performance across all hybrid systems. The transition from single-layer to double-layer laminates results in tensile strength increases on the order of 80–100%, with the most pronounced relative improvement observed in CG hybrids. This trend suggests that the additional layer contributes to improved stress redistribution and suppression of premature crack initiation under tensile loading. When normalized by density, double-layer AC laminates exhibit the highest specific tensile strength, indicating that carbon–aramid hybridization provides the most efficient strength-to-weight performance among the investigated configurations (Figure 16). This finding is particularly relevant for externally bonded strengthening systems, where minimizing additional mass is a critical design consideration.

### 4.2. Flexural Behavior

**Figure 17:**
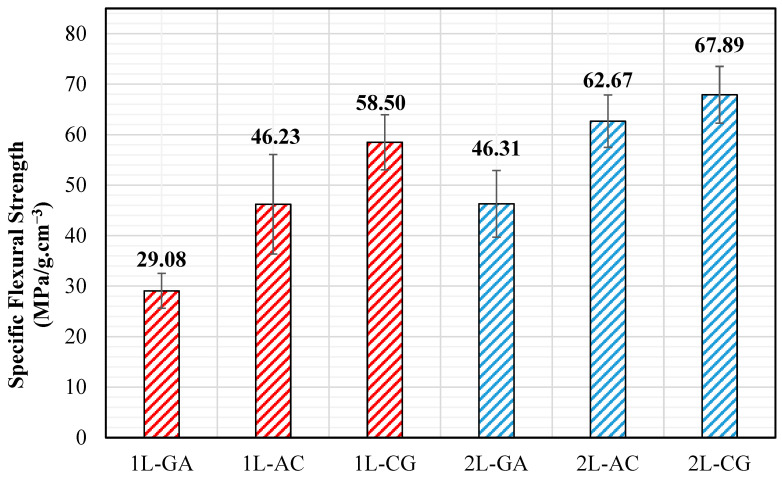
Specific Flexural Strength Values.

Flexural test results further highlight the role of intraply hybridization in governing bending performance. In single-layer form, CG laminates display the highest flexural strength, reflecting the strong influence of carbon fibers in regions subjected to tensile and compressive bending stresses. GA laminates, while exhibiting lower absolute flexural strength, show a more gradual load–displacement response, indicating enhanced energy absorption and damage tolerance. The introduction of a second layer leads to substantial improvements in flexural behavior for all hybrid systems. The 2L-CG laminate achieves the highest flexural strength (~97 MPa) and exhibits a smoother post-peak response, suggesting improved through-thickness stress continuity and reduced stress concentration effects (Figure 17). Meanwhile, 2L-AC laminates demonstrate increased stiffness and stable load redistribution, reflecting effective interaction between carbon and aramid yarns under bending. Specific flexural strength values reinforce these trends, with double-layer CG and AC laminates outperforming their single-layer counterparts by approximately 20–40%, confirming the beneficial role of layer stacking in enhancing bending efficiency under realistic fabrication conditions.

### 4.3. Compressive Behavior

**Figure 18:**
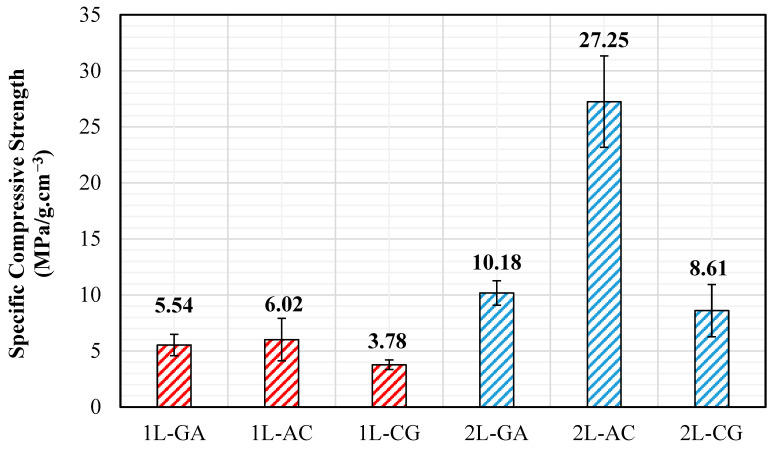
Specific Compressive Strength Values.

Compression testing, conducted in accordance with ASTM D3410/D3410M [29], reveals the most pronounced influence of layer stacking and fabrication-induced variability. Single-layer hybrids exhibit limited compressive capacity (approximately 5–8 MPa), with early instability associated with fiber waviness, resin-rich zones, and local imperfections inherent to the hand lay-up process. In contrast, double-layer laminates display a substantial increase in compressive stability. The most notable improvement is observed in the 2L-AC system, which reaches compressive strength levels on the order of 34 MPa, corresponding to a more than fivefold increase relative to its single-layer counterpart. This improvement suggests that carbon–aramid hybridization, combined with increased laminate thickness, enhances resistance to stability-controlled failure mechanisms such as microbuckling. CG and GA systems also demonstrate meaningful compressive gains (approximately two- to threefold); however, their improvements remain less pronounced than those observed for AC hybrids. Density-normalized compressive results confirm the superior efficiency of the AC configuration, with 2L-AC achieving the highest specific compressive strength among all investigated systems (Figure 18).

### 4.4. Density and Specific Performance Considerations

Density measurements reveal predictable yet informative trends. CG hybrids exhibit the highest densities due to the presence of carbon and glass fibers, whereas aramid-rich GA and AC hybrids yield lower densities. When mechanical performance is normalized by density, the classification becomes more insightful: AC hybrids consistently provide the highest specific tensile, compressive, and competitive flexural strengths, confirming that the carbon–aramid synergy delivers the most efficient combination of strength and low mass. Conversely, GA hybrids—while lower in absolute strength—demonstrate meaningful performance gains when evaluated through specific metrics, particularly in flexure, where their ductile character enables higher energy absorption per unit mass.

These findings collectively emphasize that intraply hybridization is not merely a material configuration choice but a performance-governing design strategy, enabling composite laminates to surpass the limitations of monolithic fiber systems. By integrating fibers of distinct stiffness, toughness, and failure characteristics within the same woven architecture, IRCs achieve a unique balance of strength, stiffness, ductility, and stability that cannot be replicated by single-fiber or interply hybrid laminates. This synergistic advantage is especially evident in the AC and CG systems, where carbon’s stiffness is counterbalanced by the crack-moderating role of aramid or glass, leading to enhanced load redistribution, delayed damage initiation, and more controlled failure progression. The mechanical improvements observed in double-layer hybrids further confirm the robustness of intraply designs, demonstrating that hybrid systems maintain high performance even under hand lay-up conditions characterized by imperfect consolidation.

The results collectively confirm that intraply hybridization is not merely a material configuration choice, but a performance-governing design strategy capable of tailoring mechanical response through fiber selection and layer stacking. Importantly, the observed performance trends remain consistent despite the inherent variability associated with hand lay-up fabrication, demonstrating that intraply hybrid composites retain their functional advantages under realistic field conditions. Rather than attempting to replicate idealized laboratory-quality laminates, the present study establishes a mechanical performance baseline for intraply hybrid composites as they are commonly applied in civil engineering practice. The findings therefore provide a practical framework for selecting hybrid configurations based on specific strengthening requirements, such as stiffness-dominated, ductility-oriented, or weight-sensitive applications.

### 4.5. Visual Inspection (Microscope and SEM Images)

In order to qualitatively assess the microstructural integrity and manufacturing-induced imperfections of the intraply hybrid composite laminates, detailed visual inspections were conducted using an optical microscope with a magnification capacity of up to 1600×. This examination aimed to complement the mechanical test results by identifying typical defects associated with the hand lay-up fabrication method, which is known to introduce variability in fiber impregnation, alignment, and consolidation, particularly in hybrid textile architectures.

**Figure 19:**
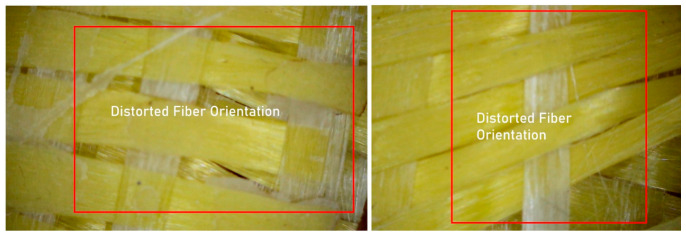
Representative Microstructural View Illustrating Distorted Fiber Orientation within the Composite Matrix.

Microscopic observations clearly revealed several recurring defect types across all laminate configurations. One of the most prominent findings was the variation in fiber orientation, which deviated locally from the intended warp–weft alignment. These misalignments are attributed to manual placement of the fabrics and localized disturbances during resin application and rolling (Figure 19). In intraply hybrid fabrics, where different fiber types coexist within the same woven layer, such orientation irregularities can lead to non-uniform stress transfer between adjacent yarns and contribute to localized stiffness mismatches under mechanical loading.

**Figure 20:**
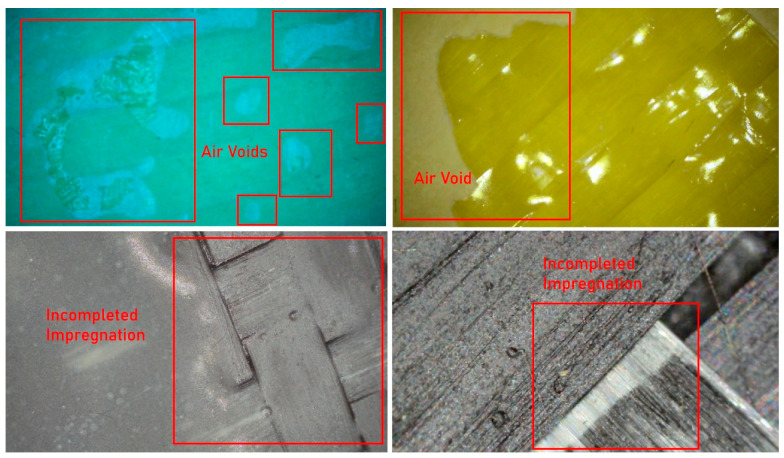
Microstructural Evidence of Incomplete Impregnation of Fiber Bundles by the Epoxy Matrix.

**Figure 21:**
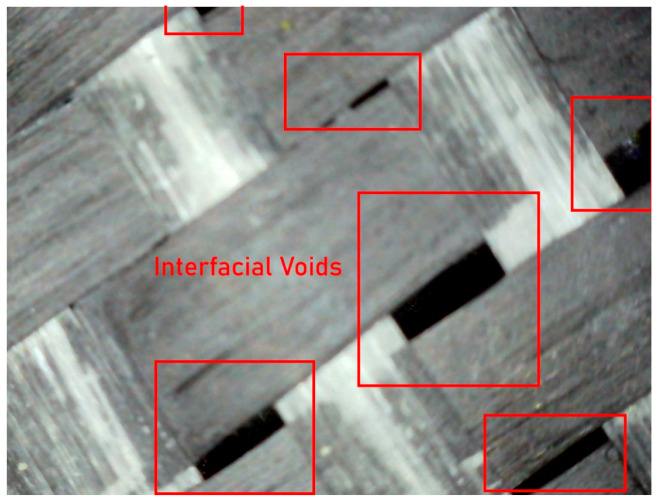
Presence of Voids within the Epoxy Matrix and at the Fiber–Matrix Interface.

**Figure 22:**
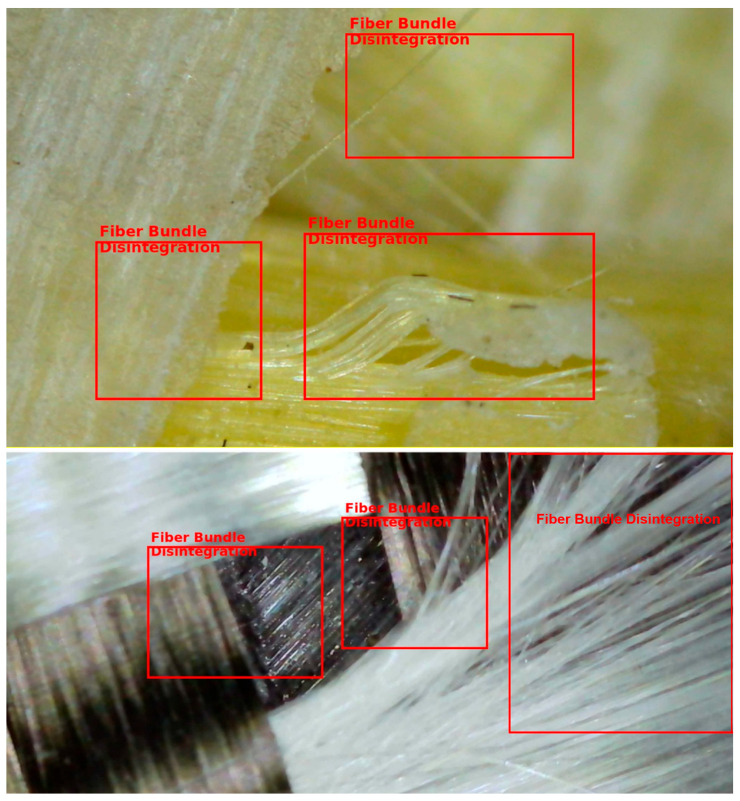
Localized Fiber Bundle Disintegration.

**Figure 23:**
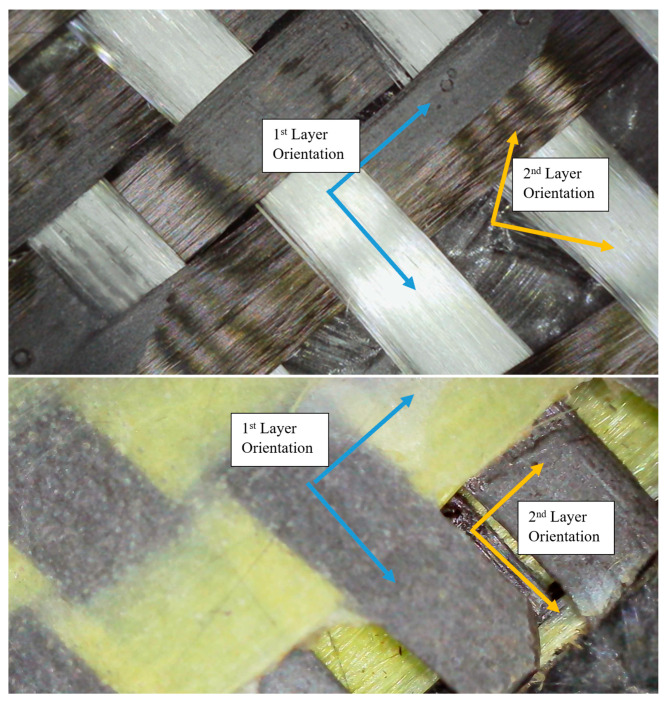
Misalignment Between the First and Second Reinforcement Layers Leading to Interlayer Discontinuities.

Another frequently observed feature was the presence of resin-starved regions, where fiber bundles were not fully impregnated by the epoxy matrix. Due to the relatively low surface energy and resin-repellent nature of aramid fibers, incomplete wet-out was locally unavoidable under non-vacuum conditions (Figure 20). These dry or partially wetted zones act as weak interfaces, promoting early matrix cracking and fiber–matrix debonding, especially under tensile and flexural stresses. In addition, entrapped air bubbles and micro-voids were widely detected within the matrix-rich regions. These voids ranged from isolated spherical bubbles to interconnected porous zones, reflecting insufficient air evacuation during manual impregnation (Figure 21). Such voids are a well-documented characteristic of hand lay-up composites and are known to reduce effective load-bearing area, degrade interfacial bonding, and act as crack initiation sites under both static and cyclic loading. Microscopic images also highlighted filament separation and local fiber bundle disintegration, particularly in regions where excessive resin accumulation occurred. In these areas, fiber filaments were observed to lose their cohesive arrangement, resulting in highly porous microstructures (Figure 22). This phenomenon was more pronounced in laminates containing aramid fibers, where differential resin absorption between adjacent fiber types promoted local heterogeneity in fiber packing density. For double-layer laminates, an additional defect mechanism was identified: misalignment between the first and second layers. In several specimens, the two intraply layers were not perfectly aligned in the same principal direction, leading to angular offsets between corresponding yarn systems (Figure 23). This layer-to-layer orientation mismatch introduces localized interlayer shear stresses and can partially explain the scatter observed in compressive and flexural test results. While such misalignment did not prevent significant strength gains in double-layer systems, it represents an inherent limitation of manual stacking without mechanical guides or vacuum consolidation.

Overall, the visual inspection results confirm that the hand lay-up method inherently introduces a range of microstructural imperfections, including fiber misorientation, incomplete resin impregnation, air voids, filament separation, and interlayer misalignment. Importantly, these defects were consistently observed across all hybrid configurations, indicating that the comparative mechanical trends reported in this study are governed primarily by hybrid fiber architecture and layer stacking, rather than by isolated manufacturing anomalies. The microscopic evidence therefore supports the central premise of this research: intraply hybrid composites retain their functional advantages and predictable performance characteristics even under realistic, field-representative fabrication conditions, where ideal consolidation cannot be fully achieved.

**Figure 24:**
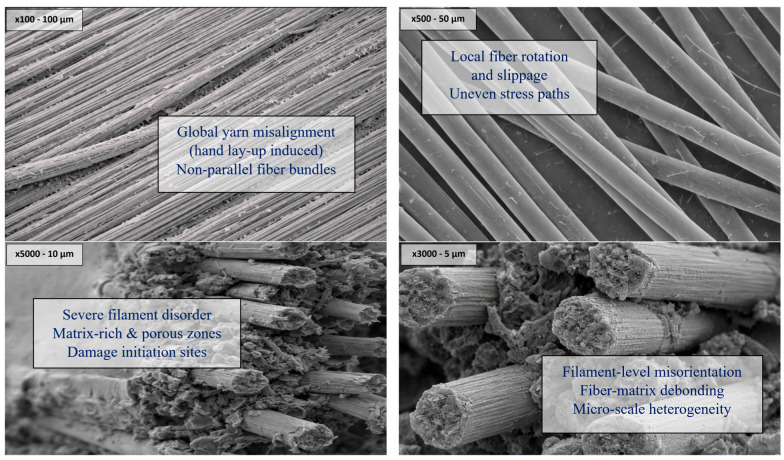
SEM Images obtained from the IRCs.

In addition to optical microscopy, SEM investigations were performed to further elucidate the microstructural characteristics of the intraply hybrid composite laminates at higher spatial resolutions. SEM observations of the fibers were carried out using a COXEM EM-40 Tabletop Scanning Electron Microscope (COXEM Co., Ltd., Daejeon, Republic of Korea). The analyses were performed under high-vacuum conditions with an accelerating voltage of 5 kV. Secondary electron imaging (SEI) mode was employed to examine the surface morphology and topographical features of the fibers. Prior to imaging, the fiber samples were sputter-coated with a thin gold layer to enhance surface conductivity and improve image resolution. SEM imaging enabled a more detailed examination of fiber–matrix interactions, local fiber orientation changes, and matrix continuity that could not be fully resolved through optical methods alone. For this purpose, representative regions were imaged at magnifications corresponding to characteristic length scales of approximately 100 μm, 50 μm, 10 μm, and 5 μm, allowing a multi-scale assessment of the manufacturing-induced microstructural features (Figure 24).

SEM observations at the 100 μm and 50 μm scales clearly confirmed the presence of non-uniform fiber orientations within the woven architecture. While the global warp–weft pattern remained identifiable, local deviations in yarn trajectories were frequently observed, particularly near resin-rich regions and at fiber crossover points. These deviations are directly attributed to the hand lay-up process, where manual handling, fabric draping, and roller compaction can induce localized fiber slippage and rotation prior to full resin gelation. In intraply hybrid fabrics, this effect was amplified by the coexistence of fibers with different stiffnesses and surface characteristics, leading to differential resistance to in-plane movement during impregnation.

At higher magnifications of 10 μm and 5 μm, SEM images revealed more subtle but mechanically relevant features associated with fiber orientation disturbance. Individual filaments within yarn bundles exhibited noticeable angular dispersion rather than maintaining a parallel alignment. This filament-level misorientation is indicative of shear-induced rearrangement during manual resin application and rolling, where localized pressure gradients can disrupt the original fiber packing. Such micro-scale orientation variability alters the effective load transfer path between the matrix and fibers, particularly under tensile and flexural loading, where stress is preferentially carried along the fiber direction. Furthermore, SEM analysis provided clear evidence of localized fiber–matrix debonding and interfacial gaps in regions where fiber orientation changes were most pronounced. These interfacial imperfections were frequently observed adjacent to aramid fibers, consistent with their lower surface wettability compared to carbon or glass fibers. The combination of partial wet-out and filament misalignment resulted in heterogeneous stress concentrations at the microscale, which likely contributed to the progressive damage mechanisms observed during mechanical testing, rather than abrupt brittle failure. Overall, the SEM findings corroborate the optical microscopy observations by demonstrating that fiber orientation variability is a pervasive and multi-scale characteristic of hand lay-up intraply hybrid composites. Importantly, these orientation changes were not random defects but systematic consequences of realistic, field-representative fabrication conditions. The consistency of these features across different magnification levels reinforces the interpretation that the mechanical behavior of the laminates is governed by an interplay between hybrid fiber architecture and manufacturing-induced microstructural heterogeneity. Consequently, the SEM evidence further supports the robustness of the comparative performance trends discussed in this study, highlighting that intraply hybrid composites can sustain predictable mechanical responses even in the presence of unavoidable microstructural imperfections inherent to manual fabrication techniques.

### 4.6. Statistical Analysis

A one-way analysis of variance (ANOVA) was performed to statistically assess the significance of differences observed among the composite configurations for tensile strength, Young’s modulus, flexural strength, compressive strength, and density (Supplementary File S1). Each experimental group consisted of five specimens (*n* = 5), and the significance level was set to α = 0.05. The analyzed groups corresponded to the six composite configurations: 1L-GA, 1L-AC, 1L-CG, 2L-GA, 2L-AC, and 2L-CG.

One-way ANOVA evaluates whether the mean values of a response variable differ significantly among multiple groups by comparing the variance between groups to the variance within groups. The *F*-statistic is defined as:

$$F=\frac{MS_{\mathrm{between}}}{MS_{\mathrm{within}}}$$

where $MS_{\mathrm{between}}$ is the mean square between groups and $MS_{\mathrm{within}}$ is the mean square within groups. For the two-way ANOVA, the total variability is decomposed into contributions from the main factors (composite type and layer number) and their interaction:

$$SS_{\mathrm{total}}=SS_{\mathrm{type}}+SS_{\mathrm{layer}}+SS_{\mathrm{type}\times \mathrm{layer}}+SS_{\mathrm{error}}$$

This formulation enables the identification of whether the influence of one factor depends on the level of the other factor.

All investigated properties exhibited highly significant differences among the composite configurations (*p* < 0.001). The extremely large *F*-values, particularly for tensile and compressive strength, indicate that the variability introduced by composite configuration overwhelmingly exceeds the experimental scatter within each group. Effect size analysis further supports this observation. For tensile strength, the eta-squared value exceeded 0.99, indicating that more than 99% of the total variance is explained by differences among configurations, which reflects a very strong structural dependency on reinforcement strategy. Post hoc Tukey HSD tests revealed that almost all pairwise comparisons were statistically significant, confirming that both fiber type and layer number contribute to distinct mechanical response levels rather than forming overlapping performance clusters. The calculated *F*-statistics and corresponding *p*-values obtained from the experimental datasets are summarized in Table 3.

To decouple the influence of material composition and reinforcement layout, a two-way ANOVA was performed. The two-way ANOVA results clearly demonstrate that composite type and layer number are both statistically significant governing parameters for all mechanical properties investigated (Table 4, Supplementary File S2). In particular, tensile and compressive strengths show strong interaction effects, indicating that the performance gain associated with increasing the number of layers is not uniform across different fiber systems. In contrast, the interaction term for flexural strength was not statistically significant (*p* = 0.777), suggesting that the flexural performance enhancement provided by the second layer is largely independent of fiber type. This behavior implies a bending-dominated response mechanism primarily controlled by global stiffness rather than fiber-specific synergy. For density, although both main factors were significant, the interaction effect was statistically insignificant. This confirms that density variations are mainly additive and do not scale synergistically with layer number, which is consistent with the observed mechanical performance gains occurring without a proportional increase in material weight.

From an engineering standpoint, the statistical analyses confirm that the transition from single-layer to double-layer reinforcement constitutes a dominant performance driver, particularly for tensile and compressive loading conditions. Moreover, the statistically significant interaction effects highlight that hybrid composite design cannot be optimized solely based on material selection or layer number independently, but rather through their combined consideration.

The experimental scatter and repeatability of the results were quantified using standard deviation and coefficient of variation (CoV). Table 5 summarizes the mean values, standard deviations, and CoV for tensile, flexural, compressive, and density measurements. The CoV values generally remain within the range expected for hand lay-up manufactured composites, confirming that configuration-driven performance differences dominate over manufacturing-induced variability.

**Figure 25:**
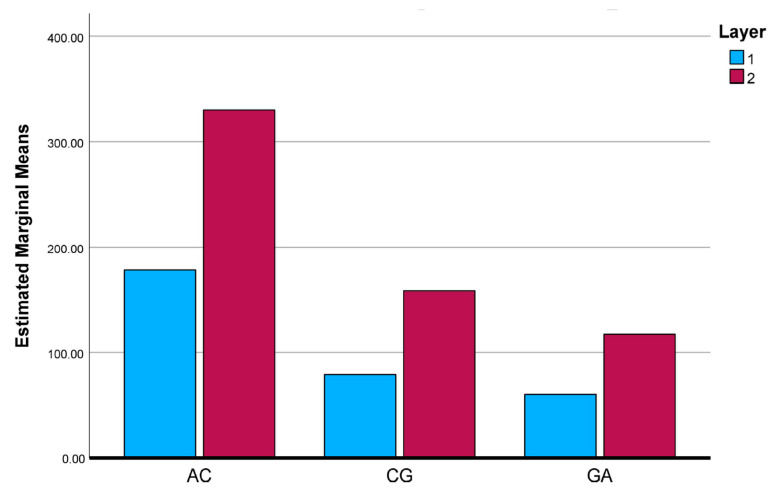
Estimated Marginal Means of Tensile Test.

**Figure 26:**
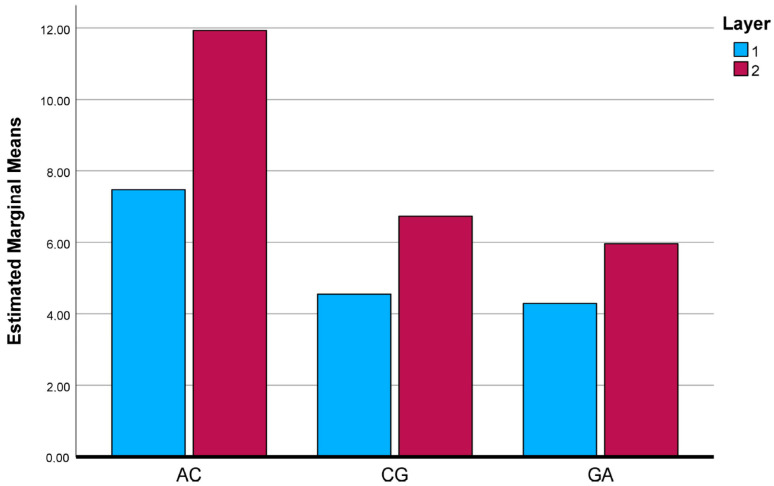
Estimated Marginal Means of Young ModuIus.

**Figure 27:**
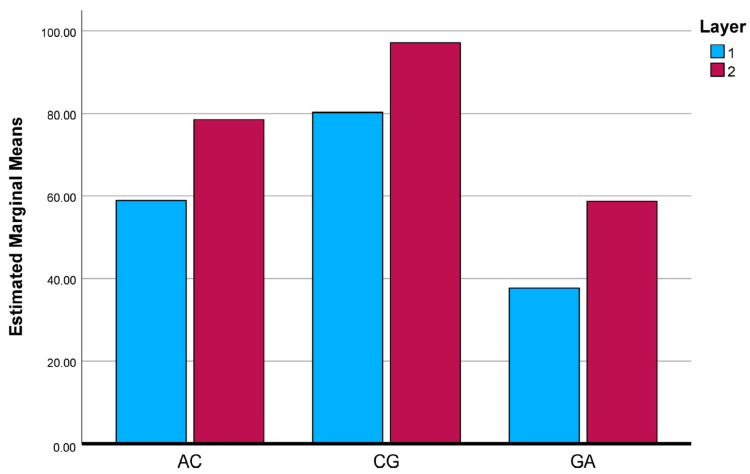
Estimated Marginal Means of Flexural Strength Test.

**Figure 28:**
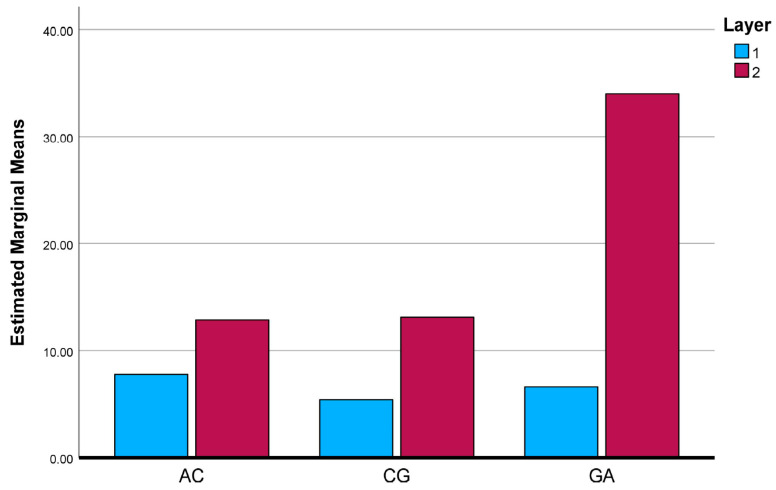
Estimated Marginal Means of Compressive Strength Test.

**Figure 29:**
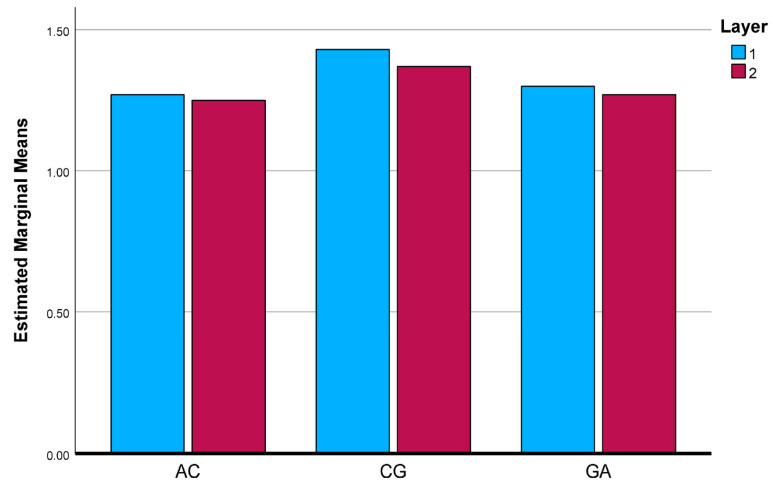
Estimated Marginal Means of Density Test.

To further clarify the interaction effects identified by the two-way ANOVA, the estimated marginal means (EMMs) of the investigated mechanical and physical properties were evaluated and graphically illustrated. Figure 25, Figure 26, Figure 27, Figure 28 and Figure 29 present the EMM plots for tensile strength, Young’s modulus, flexural strength, compressive strength, and density, respectively. These figures provide a direct visualization of how the effect of layer number varies across different composite types, thereby complementing the statistical findings obtained from the ANOVA analyses.

### 4.7. Interaction Between Laminate Strength, Scale Effect, and Manufacturing Imperfections

The experimental results highlight a strong interaction between laminate strength, scale effect associated with the number of intraply layers, and manufacturing technology imperfections inherent to the hand lay-up process. Single-layer laminates exhibit greater sensitivity to local defects such as micro-voids, resin-rich regions, and incomplete fiber impregnation, as reflected by higher scatter in flexural and compressive responses. In these configurations, localized imperfections directly govern damage initiation and early failure.

Increasing the laminate scale from single- to double-layer configurations leads to a marked improvement in mechanical performance across all loading modes. This enhancement is not solely attributed to increased thickness, but also to improved stress redistribution and structural redundancy, which mitigate the influence of localized manufacturing defects. As a result, double-layer laminates demonstrate higher strength, delayed damage evolution, and reduced result variability.

The observed behavior confirms that, under hand lay-up conditions, manufacturing imperfections primarily affect single-layer laminates, whereas scale effects associated with additional layers progressively suppress defect sensitivity. Consequently, laminate architecture and layer number emerge as dominant performance drivers, outweighing random imperfections introduced by the fabrication process. This finding is particularly relevant for field applications, where perfect manufacturing control is rarely achievable.

## 5. Conclusions

This study investigated the mechanical behavior of intraply hybrid composite laminates manufactured using the hand lay-up technique, focusing on three hybrid fabric configurations: Glass–Aramid (GA), Aramid–Carbon (AC), and Carbon–Glass (CG). Tensile, flexural, compressive, and density-related experimental results were evaluated to clarify the role of fiber hybridization and layer stacking under field-representative manufacturing conditions relevant to structural strengthening applications.

Based on the experimental investigation of single- and double-layer intraply hybrid composite laminates manufactured by the hand lay-up method, the following key conclusions can be drawn:

Intraply hybridization effectively tailors mechanical response under realistic, field-representative manufacturing conditions. Despite the inherent variability of hand lay-up fabrication, all hybrid systems exhibited consistent and distinguishable mechanical performance trends.

Aramid–Carbon (AC) hybrids provided the highest overall mechanical efficiency, achieving the greatest tensile stiffness, tensile strength, and compressive resistance. Double-layer AC laminates demonstrated the best strength-to-weight performance among all configurations.

Carbon–Glass (CG) hybrids exhibited superior flexural performance, particularly in double-layer form, indicating that this hybrid configuration benefits most from layer stacking under bending-dominated loading conditions.

Glass–Aramid (GA) hybrids showed lower stiffness and strength but higher deformation capacity, highlighting their suitability for applications where ductility and progressive failure behavior are preferred over peak load capacity.

Layer stacking (single- to double-layer transition) was identified as a dominant performance driver, leading to significant improvements in tensile, flexural, and compressive properties by enhancing stress redistribution and suppressing defect sensitivity.

Compressive performance was strongly governed by laminate scale and stability effects, with double-layer laminates—especially AC hybrids—exhibiting markedly enhanced resistance to microbuckling and instability-controlled failure.

Density-normalized (specific) properties confirmed that hybrid architecture, rather than absolute material mass, governs performance efficiency, with AC systems consistently achieving the highest specific strengths.

Overall, the results demonstrate that IRCs retain their functional advantages under non-ideal manufacturing conditions, establishing a reliable mechanical performance baseline for their use in on-site structural strengthening and rehabilitation applications.

Future studies should therefore expand specimen populations, incorporate microstructural and durability analyses, explore alternative matrix systems, and include comparative investigations using vacuum-assisted or pressure-controlled manufacturing techniques. Such efforts would further enhance understanding of intraply hybrid composite behavior and support the development of predictive design frameworks for structural engineering applications.

## Figures and Tables

**Table 1:** Properties of Teknobond 300 TIX epoxy adhesive (manufacturer’s datasheet).

Property	Value/Description
Type	Two-component epoxy adhesive
Mixing ratio (A:B, by weight)	5.80:1.40
Density	~1.20 g/cm^3^
Viscosity (25 °C)	1500–2500 mPa·s
Pot life (25 °C)	40–60 min
Curing time (25 °C)	7 days
Tensile strength	30–35 MPa
Flexural strength	60–70 MPa
Bonding strength to concrete	>3.0 MPa

**Table 2:** Specimen geometry and applicable ASTM standards.

Test Type	ASTM Standard	Key Dimensional Requirements
Tensile Test	ASTM D3039/D3039M [27]	Nominal length: 250 mm; width: 25 mm; gauge length: 150 mm; thickness: as-fabricated laminate thickness
Flexural Test (3-point bending)	ASTM D790 [28]	Nominal width: 12.7 mm; length adjusted to maintain span-to-thickness ratio of 16:1; thickness: as-fabricated laminate thickness
Compressive Test	ASTM D3410/D3410M [29]	Nominal length: 140 mm; width: 13 mm; thickness: as-fabricated laminate thickness
Density Measurement	ASTM D792 [30]	Dimensions selected to ensure representative volume; density calculated from measured mass and volume

**Table 3:** One-way ANOVA results for mechanical and physical properties of the composite configurations.

Property	df (Between)	df (Within)	*F*-Value	*p*-Value
Tensile strength	5	24	535.23	<0.001
Young’s modulus	5	24	104.80	<0.001
Flexural strength	5	24	53.15	<0.001
Compressive strength	5	24	189.80	<0.001
Density	5	24	66.00	<0.001

**Table 4:** Two-way ANOVA results for composite type and layer number.

Property	Factor	df	*F*-Value	*p*-Value
Tensile strength	Composite type	2	875.79	<0.001
	Layer number	1	785.52	<0.001
	Type × Layer	2	69.53	<0.001
Young’s modulus	Composite type	2	169.95	<0.001
	Layer number	1	154.76	<0.001
	Type × Layer	2	14.66	<0.001
Flexural strength	Composite type	2	99.27	<0.001
	Layer number	1	66.71	<0.001
	Type × Layer	2	0.25	0.777
Compressive strength	Composite type	2	123.98	<0.001
	Layer number	1	453.09	<0.001
	Type × Layer	2	123.98	<0.001
Density	Composite type	2	148.67	<0.001
	Layer number	1	26.89	<0.001
	Type × Layer	2	2.89	0.075

**Table 5:** Mechanical and Physical Properties with Variability Measures.

Configuration	Property	Mean ± SD	CoV (%)
1L-GA	Tensile Strength (MPa)	60.22 ± 3.73	6.2
	Young’s Modulus (GPa)	4.29 ± 0.30	6.9
	Flexural Strength (MPa)	37.80 ± 3.13	8.3
	Compressive Strength (MPa)	6.61 ± 0.69	10.5
	Density (g/cm^3^)	1.30 ± 0.022	1.7
1L-AC	Tensile Strength (MPa)	178.18 ± 5.93	3.3
	Young’s Modulus (GPa)	7.49 ± 0.51	6.8
	Flexural Strength (MPa)	58.88 ± 9.59	16.3
	Compressive Strength (MPa)	7.77 ± 0.56	7.2
	Density (g/cm^3^)	1.27 ± 0.016	1.2
1L-CG	Tensile Strength (MPa)	79.06 ± 5.05	6.4
	Young’s Modulus (GPa)	4.55 ± 0.35	7.7
	Flexural Strength (MPa)	80.25 ± 5.65	7.0
	Compressive Strength (MPa)	5.41 ± 0.47	8.7
	Density (g/cm^3^)	1.43 ± 0.022	1.6
2L-GA	Tensile Strength (MPa)	117.30 ± 7.18	6.1
	Young’s Modulus (GPa)	5.96 ± 0.35	5.9
	Flexural Strength (MPa)	58.68 ± 6.75	11.5
	Compressive Strength (MPa)	12.91 ± 1.01	7.8
	Density (g/cm^3^)	1.27 ± 0.016	1.2
2L-AC	Tensile Strength (MPa)	330.40 ± 18.66	5.6
	Young’s Modulus (GPa)	11.93 ± 1.16	9.7
	Flexural Strength (MPa)	78.55 ± 5.44	6.9
	Compressive Strength (MPa)	34.01 ± 3.86	11.3
	Density (g/cm^3^)	1.25 ± 0.016	1.3
2L-CG	Tensile Strength (MPa)	158.94 ± 7.61	4.8
	Young’s Modulus (GPa)	6.73 ± 0.53	7.9
	Flexural Strength (MPa)	97.14 ± 6.20	6.4
	Compressive Strength (MPa)	13.16 ± 0.99	7.5
	Density (g/cm^3^)	1.37 ± 0.022	1.6

## Data Availability

Data is contained within the article or Supplementary Material.

## Supplementary Materials

The following supporting information can be downloaded at: https://www.mdpi.com/article/10.3390/polym18020188/s1. Supplementary File S1: One way ANOVA results; Supplementary File S2: Two way ANOVA results.
