# Supplementary material for: Determination of Mechanical Properties of Single and Double-Layer Intraply Hybrid Composites Manufactured by Hand Lay-Up Method
Source: Polymers (Basel). 2026 Jan 9;18(2):188. doi: 10.3390/polym18020188 (PMC12845855; doi:10.3390/polym18020188)
Supplement: Supplementary file 1 [file polymers-18-00188-s001.zip › Supplementary File S2-Two way ANOVA.pdf]

Univariate Analysis of Variance

Between-Subjects Factors

		Value Label	N
Composite_type_code	1	AC	10
	2	CG	10
	3	GA	10
Layer	1		15
	2		15

Tests of Between-Subjects Effects

Dependent Variable: Tensile_test

Source	Type III Sum of Squares	df	Mean Square	F	Sig.
Corrected Model	237421,834[a]	5	47484,367	535,233	<.001
Intercept	711627,848	1	711627,848	8021,307	<.001
Composite_type_code	155396,019	2	77698,009	875,794	<.001
Layer	69689,488	1	69689,488	785,524	<.001
Composite_type_code * Layer	12336,326	2	6168,163	69,526	<.001
Error	2129,213	24	88,717		
Total	951178,894	30			
Corrected Total	239551,046	29			

a. R Squared = ,991 (Adjusted R Squared = ,989)

Post Hoc Tests

Composite_type_code

Multiple Comparisons

Dependent Variable: Tensile_test
Tukey HSD

(I) Composite_type_code	(J) Composite_type_code	Mean Difference (I-J)	Std. Error	Sig.
AC	CG	135,2900*	4,21230	<.001
	GA	165,5320*	4,21230	<.001
CG	AC	-135,2900*	4,21230	<.001
	GA	30,2420*	4,21230	<.001
GA	AC	-165,5320*	4,21230	<.001
	CG	-30,2420*	4,21230	<.001

Multiple Comparisons

Dependent Variable: Tensile_test

Tukey HSD

(I) Composite_type_code	(J) Composite_type_code	95% Confidence Interval	
		Lower Bound	Upper Bound
AC	CG	124,7707	145,8093
	GA	155,0127	176,0513
CG	AC	-145,8093	-124,7707
	GA	19,7227	40,7613
GA	AC	-176,0513	-155,0127
	CG	-40,7613	-19,7227

Based on observed means.
The error term is Mean Square(Error) = 88,717.

*. The mean difference is significant at the 0.05 level.

Homogeneous Subsets

Tensile_test

Tukey HSD[a,b]

Composite_type_code	N	Subset		
		1	2	3
GA	10	88,7580		
CG	10		119,0000	
AC	10			254,2900
Sig.		1,000	1,000	1,000

Means for groups in homogeneous subsets are displayed.
Based on observed means.
The error term is Mean Square(Error) = 88,717.

a. Uses Harmonic Mean Sample Size = 10,000.

b. Alpha = 0.05.

Profile Plots

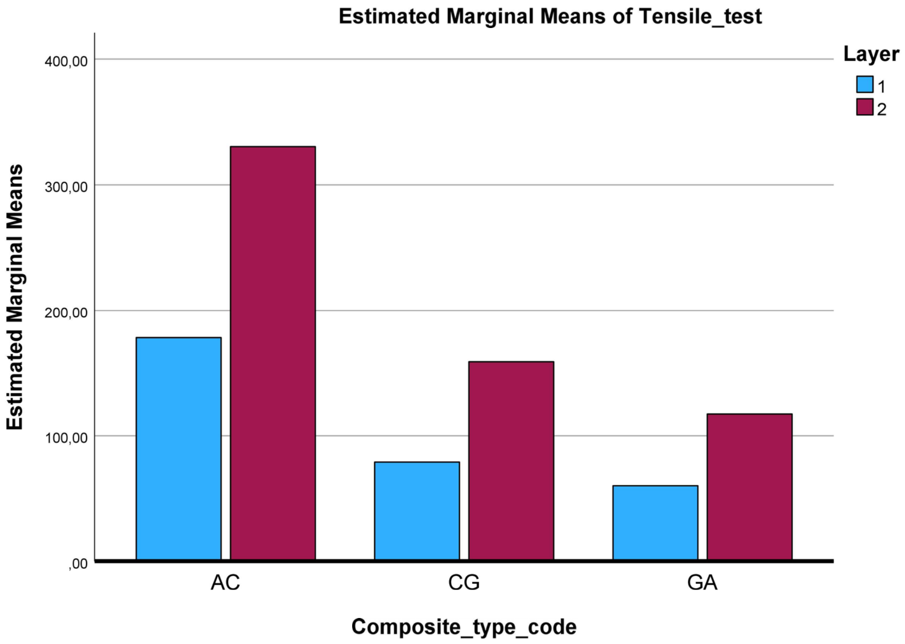

Univariate Analysis of Variance

Between-Subjects Factors

		Value Label	N
Composite_type_code	1	AC	10
	2	CG	10
	3	GA	10
Layer	1		15
	2		15

Tests of Between-Subjects Effects

Dependent Variable: Young_Modulus

Source	Type III Sum of Squares	df	Mean Square	F	Sig.
Corrected Model	194,364[a]	5	38,873	104,795	<.001
Intercept	1397,009	1	1397,009	3766,133	<.001
Composite_type_code	126,083	2	63,041	169,950	<.001
Layer	57,408	1	57,408	154,764	<.001
Composite_type_code * Layer	10,873	2	5,437	14,656	<.001
Error	8,903	24	,371		
Total	1600,276	30			
Corrected Total	203,267	29			

a. R Squared = ,956 (Adjusted R Squared = ,947)

Post Hoc Tests

Composite_type_code

Multiple Comparisons

Dependent Variable: Young_Modulus
Tukey HSD

(I) Composite_type_code	(J) Composite_type_code	Mean Difference (I-J)	Std. Error	Sig.
AC	CG	4,0690*	,27237	<.001
	GA	4,5830*	,27237	<.001
CG	AC	-4,0690*	,27237	<.001
	GA	,5140	,27237	,164
GA	AC	-4,5830*	,27237	<.001
	CG	-,5140	,27237	,164

Multiple Comparisons

Dependent Variable: Young_Modulus
Tukey HSD

(I) Composite_type_code	(J) Composite_type_code	95% Confidence Interval	
		Lower Bound	Upper Bound
AC	CG	3,3888	4,7492
	GA	3,9028	5,2632
CG	AC	-4,7492	-3,3888
	GA	-,1662	1,1942
GA	AC	-5,2632	-3,9028
	CG	-1,1942	,1662

Based on observed means.
The error term is Mean Square(Error) = ,371.
*. The mean difference is significant at the 0.05 level.

Homogeneous Subsets

Young_Modulus

Tukey HSD[a,b]

Composite_type_code	N	Subset	
		1	2
GA	10	5,1250	
CG	10	5,6390	
AC	10		9,7080
Sig.		,164	1,000

Means for groups in homogeneous subsets are displayed.
Based on observed means.
The error term is Mean Square(Error) = ,371.
a. Uses Harmonic Mean Sample Size = 10,000.
b. Alpha = 0.05.

Profile Plots

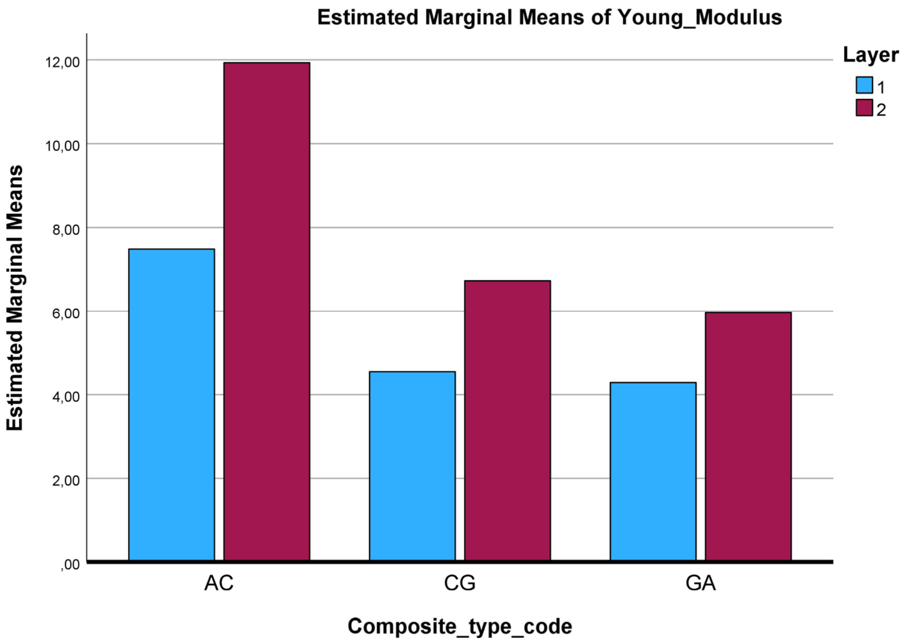

Univariate Analysis of Variance

Between-Subjects Factors

		Value Label	N
Composite_type_code	1	AC	10
	2	CG	10
	3	GA	10
Layer	1		15
	2		15

Tests of Between-Subjects Effects

Dependent Variable: Flexural_strength_test

Source	Type III Sum of Squares	df	Mean Square	F	Sig.
Corrected Model	10952,472[a]	5	2190,494	53,153	<.001
Intercept	140974,446	1	140974,446	3420,795	<.001
Composite_type_code	8182,228	2	4091,114	99,272	<.001
Layer	2749,270	1	2749,270	66,712	<.001
Composite_type_code * Layer	20,974	2	10,487	,254	,777
Error	989,065	24	41,211		
Total	152915,983	30			
Corrected Total	11941,537	29			

a. R Squared = ,917 (Adjusted R Squared = ,900)

Post Hoc Tests

Composite_type_code

Multiple Comparisons

Dependent Variable: Flexural_strength_test
Tukey HSD

(I) Composite_type_code	(J) Composite_type_code	Mean Difference (I-J)	Std. Error	Sig.
AC	CG	-19,9790*	2,87092	<.001
	GA	20,4730*	2,87092	<.001
CG	AC	19,9790*	2,87092	<.001
	GA	40,4520*	2,87092	<.001
GA	AC	-20,4730*	2,87092	<.001
	CG	-40,4520*	2,87092	<.001

Multiple Comparisons

Dependent Variable: Flexural_strength_test
Tukey HSD

(I) Composite_type_code	(J) Composite_type_code	95% Confidence Interval	
		Lower Bound	Upper Bound
AC	CG	-27,1485	-12,8095
	GA	13,3035	27,6425
CG	AC	12,8095	27,1485
	GA	33,2825	47,6215
GA	AC	-27,6425	-13,3035
	CG	-47,6215	-33,2825

Based on observed means.
The error term is Mean Square(Error) = 41,211.

*. The mean difference is significant at the 0.05 level.

Homogeneous Subsets

Flexural_strength_test

Tukey HSD[a,b]

Composite_type_code	N	Subset 1	2	3
GA	10	48,2420		
AC	10		68,7150	
CG	10			88,6940
Sig.		1,000	1,000	1,000

Means for groups in homogeneous subsets are displayed.
Based on observed means.
The error term is Mean Square(Error) = 41,211.

a. Uses Harmonic Mean Sample Size = 10,000.

b. Alpha = 0.05.

Profile Plots

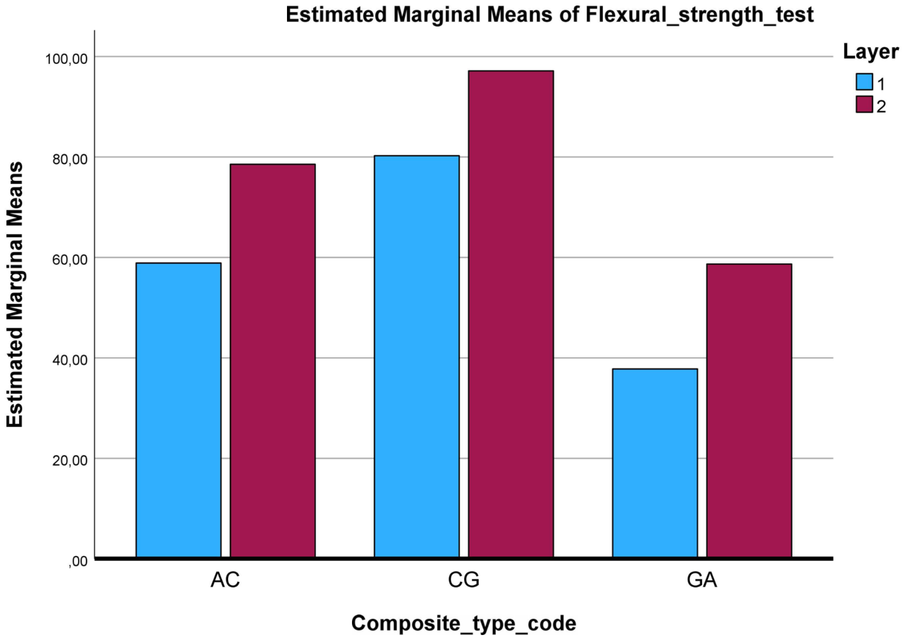

Univariate Analysis of Variance

Between-Subjects Factors

		Value Label	N
Composite_type_code	1	AC	10
	2	CG	10
	3	GA	10
Layer	1		15
	2		15

Tests of Between-Subjects Effects

Dependent Variable: Compressive_strength_test

Source	Type III Sum of Squares	df	Mean Square	F	Sig.
Corrected Model	2833,387[a]	5	566,677	189,803	<.001
Intercept	5317,079	1	5317,079	1780,907	<.001
Composite_type_code	740,334	2	370,167	123,984	<.001
Layer	1352,737	1	1352,737	453,087	<.001
Composite_type_code * Layer	740,316	2	370,158	123,981	<.001
Error	71,654	24	2,986		
Total	8222,121	30			
Corrected Total	2905,042	29			

a. R Squared = ,975 (Adjusted R Squared = ,970)

Post Hoc Tests

Composite_type_code

Multiple Comparisons

Dependent Variable: Compressive_strength_test

Tukey HSD

(I) Composite_type_code	(J) Composite_type_code	Mean Difference (I-J)	Std. Error	Sig.
AC	CG	1,0530	,77274	,376
	GA	-9,9720*	,77274	<.001
CG	AC	-1,0530	,77274	,376
	GA	-11,0250*	,77274	<.001
GA	AC	9,9720*	,77274	<.001
	CG	11,0250*	,77274	<.001

Multiple Comparisons

Dependent Variable: Compressive_strength_test

Tukey HSD

(I) Composite_type_code	(J) Composite_type_code	95% Confidence Interval Lower Bound	95% Confidence Interval Upper Bound
AC	CG	-,8767	2,9827
	GA	-11,9017	-8,0423
CG	AC	-2,9827	,8767
	GA	-12,9547	-9,0953
GA	AC	8,0423	11,9017
	CG	9,0953	12,9547

Based on observed means.
The error term is Mean Square(Error) = 2,986.

*. The mean difference is significant at the 0.05 level.

Homogeneous Subsets

Compressive_strength_test

Tukey HSD[a,b]

Composite_type_code	N	Subset	
		1	2
CG	10	9,2870	
AC	10	10,3400	
GA	10		20,3120
Sig.		,376	1,000

Means for groups in homogeneous subsets are displayed.
Based on observed means.
The error term is Mean Square(Error) = 2,986.

a. Uses Harmonic Mean Sample Size = 10,000.

b. Alpha = 0.05.

Profile Plots

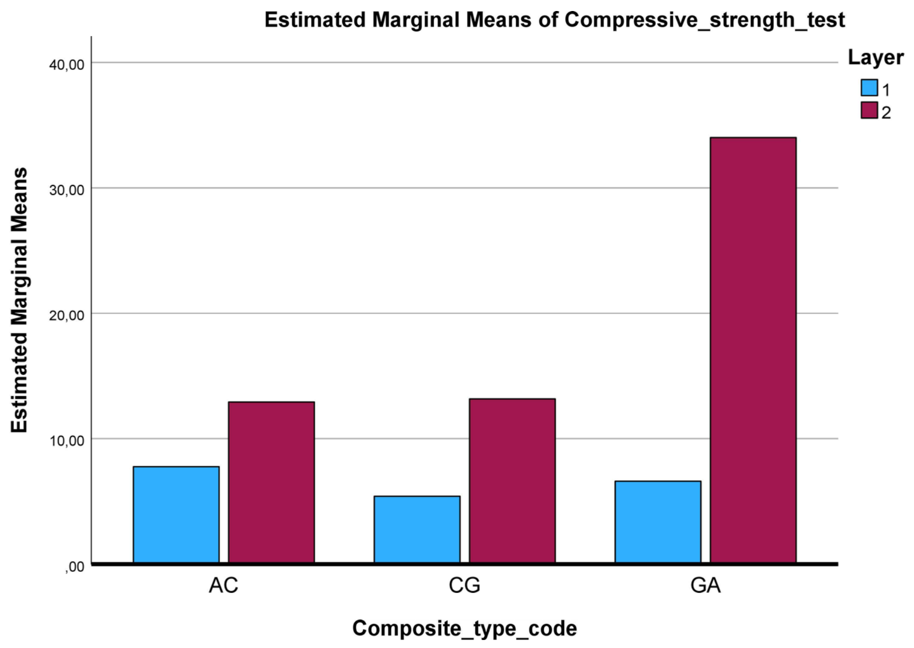

Univariate Analysis of Variance

Between-Subjects Factors

		Value Label	N
Composite_type_code	1	AC	10
	2	CG	10
	3	GA	10
Layer	1		15
	2		15

Tests of Between-Subjects Effects

Dependent Variable: Density_test

Source	Type III Sum of Squares	df	Mean Square	F	Sig.
Corrected Model	,124[a]	5	,025	66,000	<.001
Intercept	51,877	1	51,877	138338,000	<.001
Composite_type_code	,111	2	,056	148,667	<.001
Layer	,010	1	,010	26,889	<.001
Composite_type_code * Layer	,002	2	,001	2,889	,075
Error	,009	24	,000		
Total	52,010	30			
Corrected Total	,133	29			

a. R Squared = ,932 (Adjusted R Squared = ,918)

Post Hoc Tests

Composite_type_code

Multiple Comparisons

Dependent Variable: Density_test
Tukey HSD

(I) Composite_type_code	(J) Composite_type_code	Mean Difference (I-J)	Std. Error	Sig.
AC	CG	-,1400*	,00866	<.001
	GA	-,0250*	,00866	,021
CG	AC	,1400*	,00866	<.001
	GA	,1150*	,00866	<.001
GA	AC	,0250*	,00866	,021
	CG	-,1150*	,00866	<.001

Multiple Comparisons

Dependent Variable: Density_test

Tukey HSD

(I) Composite_type_code	(J) Composite_type_code	95% Confidence Interval Lower Bound	95% Confidence Interval Upper Bound
AC	CG	-,1616	-,1184
	GA	-,0466	-,0034
CG	AC	,1184	,1616
	GA	,0934	,1366
GA	AC	,0034	,0466
	CG	-,1366	-,0934

Based on observed means.
The error term is Mean Square(Error) = ,000.

*. The mean difference is significant at the 0.05 level.

Homogeneous Subsets

Density_test

Tukey HSD[a,b]

Composite_type_code	N	Subset 1	Subset 2	Subset 3
AC	10	1,2600		
GA	10		1,2850	
CG	10			1,4000
Sig.		1,000	1,000	1,000

Means for groups in homogeneous subsets are displayed.
Based on observed means.
The error term is Mean Square(Error) = ,000.

a. Uses Harmonic Mean Sample Size = 10,000.

b. Alpha = 0.05.

Profile Plots

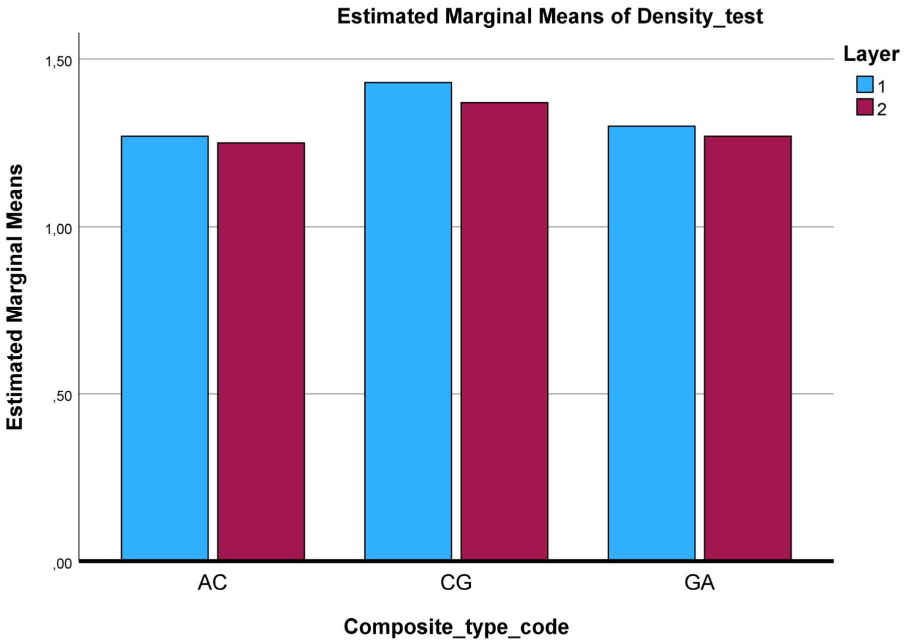
Estimated Marginal Means of Density_test
Layer
1
2
Estimated Marginal Means
1,50
1,00
,50
,00
AC
CG
GA
Composite_type_code
