# Supplementary material for: Determination of Mechanical Properties of Single and Double-Layer Intraply Hybrid Composites Manufactured by Hand Lay-Up Method
Source: Polymers (Basel). 2026 Jan 9;18(2):188. doi: 10.3390/polym18020188 (PMC12845855; doi:10.3390/polym18020188)
Supplement: Supplementary file 1 [file polymers-18-00188-s001.zip › Supplementary File S1-One way ANOVA.pdf]

Oneway

[DataSet0]

Descriptives

		N	Mean	Std. Deviation	Std. Error
Tensile_test	1L-AC-1	5	60,2160	3,73032	1,66825
	1L-AC-2	5	178,1800	5,92712	2,65069
	1L-AC-3	5	79,0600	5,04777	2,25743
	1L-AC-4	5	117,3000	7,17870	3,21041
	1L-AC-5	5	330,4000	18,66265	8,34619
	1L-CG-1	5	158,9400	7,61242	3,40438
	Total	30	154,0160	90,88664	16,59355
Young_Modulus	1L-AC-1	5	4,2880	,29752	,13306
	1L-AC-2	5	7,4860	,50639	,22646
	1L-AC-3	5	4,5480	,35174	,15730
	1L-AC-4	5	5,9620	,35429	,15844
	1L-AC-5	5	11,9300	1,16222	,51976
	1L-CG-1	5	6,7300	,52981	,23694
	Total	30	6,8240	2,64749	,48336
Flexural_strength_test	1L-AC-1	5	37,8000	3,13289	1,40107
	1L-AC-2	5	58,8820	9,58833	4,28803
	1L-AC-3	5	80,2500	5,64535	2,52468
	1L-AC-4	5	58,6840	6,75191	3,01955
	1L-AC-5	5	78,5480	5,44137	2,43346
	1L-CG-1	5	97,1380	6,20066	2,77302
	Total	30	68,5503	20,29229	3,70485
Compressive_strength_test	1L-AC-1	5	6,6120	,69424	,31047
	1L-AC-2	5	7,7680	,56291	,25174
	1L-AC-3	5	5,4140	,47125	,21075
	1L-AC-4	5	34,0120	3,85820	1,72544
	1L-AC-5	5	12,9120	1,01043	,45188
	1L-CG-1	5	13,1600	,99298	,44407
	Total	30	13,3130	10,00869	1,82733
Density_test	1L-AC-1	5	1,3000	,02236	,01000
	1L-AC-2	5	1,2700	,01581	,00707
	1L-AC-3	5	1,4300	,02236	,01000
	1L-AC-4	5	1,2700	,01581	,00707
	1L-AC-5	5	1,2500	,01581	,00707
	1L-CG-1	5	1,3700	,02236	,01000
	Total	30	1,3150	,06766	,01235

Descriptives

		95% Confidence Interval for Mean		Minimum	Maximum
		Lower Bound	Upper Bound		
Tensile_test	1L-AC-1	55,5842	64,8478	55,32	64,98
	1L-AC-2	170,8205	185,5395	170,42	185,69
	1L-AC-3	72,7924	85,3276	72,68	85,54
	1L-AC-4	108,3865	126,2135	108,24	126,36
	1L-AC-5	307,2273	353,5727	306,48	354,92
	1L-CG-1	149,4879	168,3921	149,36	168,70
	Total	120,0784	187,9536	55,32	354,92
Young_Modulus	1L-AC-1	3,9186	4,6574	3,92	4,68
	1L-AC-2	6,8572	8,1148	6,85	8,15
	1L-AC-3	4,1113	4,9847	4,12	5,02
	1L-AC-4	5,5221	6,4019	5,52	6,42
	1L-AC-5	10,4869	13,3731	10,45	13,40
	1L-CG-1	6,0722	7,3878	6,10	7,45
	Total	5,8354	7,8126	3,92	13,40
Flexural_strength_test	1L-AC-1	33,9100	41,6900	33,90	41,90
	1L-AC-2	46,9765	70,7875	47,20	71,30
	1L-AC-3	73,2404	87,2596	73,20	87,40
	1L-AC-4	50,3004	67,0676	50,10	67,20
	1L-AC-5	71,7916	85,3044	71,80	85,60
	1L-CG-1	89,4389	104,8371	89,40	105,20
	Total	60,9731	76,1276	33,90	105,20
Compressive_strength_test	1L-AC-1	5,7500	7,4740	5,72	7,48
	1L-AC-2	7,0691	8,4669	7,05	8,48
	1L-AC-3	4,8289	5,9991	4,82	6,02
	1L-AC-4	29,2214	38,8026	29,10	38,90
	1L-AC-5	11,6574	14,1666	11,62	14,18
	1L-CG-1	11,9271	14,3929	11,90	14,42
	Total	9,5757	17,0503	4,82	38,90
Density_test	1L-AC-1	1,2722	1,3278	1,27	1,33
	1L-AC-2	1,2504	1,2896	1,25	1,29
	1L-AC-3	1,4022	1,4578	1,40	1,46
	1L-AC-4	1,2504	1,2896	1,25	1,29
	1L-AC-5	1,2304	1,2696	1,23	1,27
	1L-CG-1	1,3422	1,3978	1,34	1,40
	Total	1,2897	1,3403	1,23	1,46

ANOVA

		Sum of Squares	df	Mean Square	F
Tensile_test	Between Groups	237421,834	5	47484,367	535,233
	Within Groups	2129,213	24	88,717	
	Total	239551,046	29		
Young_Modulus	Between Groups	194,364	5	38,873	104,795
	Within Groups	8,903	24	,371	
	Total	203,267	29		
Flexural_strength_test	Between Groups	10952,472	5	2190,494	53,153
	Within Groups	989,065	24	41,211	
	Total	11941,537	29		
Compressive_strength_test	Between Groups	2833,387	5	566,677	189,803
	Within Groups	71,654	24	2,986	
	Total	2905,042	29		
Density_test	Between Groups	,124	5	,025	66,000
	Within Groups	,009	24	,000	
	Total	,133	29		

ANOVA

		Sig.
Tensile_test	Between Groups	<.001
	Within Groups	
	Total	
Young_Modulus	Between Groups	<.001
	Within Groups	
	Total	
Flexural_strength_test	Between Groups	<.001
	Within Groups	
	Total	
Compressive_strength_test	Between Groups	<.001
	Within Groups	
	Total	
Density_test	Between Groups	<.001
	Within Groups	
	Total	

ANOVA Effect Sizes[a]

		Point Estimate	95% Confidence Interval Lower	Upper
Tensile_test	Eta-squared	,991	,979	,993
	Epsilon-squared	,989	,974	,992
	Omega-squared Fixed-effect	,989	,973	,992
	Omega-squared Random-effect	,947	,879	,959
Young_Modulus	Eta-squared	,956	,895	,967
	Epsilon-squared	,947	,874	,960
	Omega-squared Fixed-effect	,945	,870	,958
	Omega-squared Random-effect	,776	,572	,822
Flexural_strength_test	Eta-squared	,917	,805	,937
	Epsilon-squared	,900	,764	,924
	Omega-squared Fixed-effect	,897	,758	,922
	Omega-squared Random-effect	,635	,385	,702
Compressive_strength_test	Eta-squared	,975	,941	,981
	Epsilon-squared	,970	,928	,977
	Omega-squared Fixed-effect	,969	,926	,977
	Omega-squared Random-effect	,863	,715	,893
Density_test	Eta-squared	,932	,839	,949
	Epsilon-squared	,918	,806	,938
	Omega-squared Fixed-effect	,915	,800	,936
	Omega-squared Random-effect	,684	,445	,745

a. Eta-squared and Epsilon-squared are estimated based on the fixed-effect model.

Post Hoc Tests

Multiple Comparisons

Tukey HSD

Dependent Variable	(I) Series_code	(J) Series_code	Mean Difference (I-J)	Std. Error	Sig.
Tensile_test	1L-AC-1	1L-AC-2	-117,96400*	5,95709	<.001
		1L-AC-3	-18,84400*	5,95709	,043
		1L-AC-4	-57,08400*	5,95709	<.001
		1L-AC-5	-270,18400*	5,95709	<.001
		1L-CG-1	-98,72400*	5,95709	<.001
	1L-AC-2	1L-AC-1	117,96400*	5,95709	<.001
		1L-AC-3	99,12000*	5,95709	<.001
		1L-AC-4	60,88000*	5,95709	<.001
		1L-AC-5	-152,22000*	5,95709	<.001
		1L-CG-1	19,24000*	5,95709	,037
	1L-AC-3	1L-AC-1	18,84400*	5,95709	,043
		1L-AC-2	-99,12000*	5,95709	<.001
		1L-AC-4	-38,24000*	5,95709	<.001
		1L-AC-5	-251,34000*	5,95709	<.001
		1L-CG-1	-79,88000*	5,95709	<.001
	1L-AC-4	1L-AC-1	57,08400*	5,95709	<.001
		1L-AC-2	-60,88000*	5,95709	<.001
		1L-AC-3	38,24000*	5,95709	<.001
		1L-AC-5	-213,10000*	5,95709	<.001
		1L-CG-1	-41,64000*	5,95709	<.001
	1L-AC-5	1L-AC-1	270,18400*	5,95709	<.001
		1L-AC-2	152,22000*	5,95709	<.001
		1L-AC-3	251,34000*	5,95709	<.001
		1L-AC-4	213,10000*	5,95709	<.001
		1L-CG-1	171,46000*	5,95709	<.001
	1L-CG-1	1L-AC-1	98,72400*	5,95709	<.001
		1L-AC-2	-19,24000*	5,95709	,037
		1L-AC-3	79,88000*	5,95709	<.001
		1L-AC-4	41,64000*	5,95709	<.001
		1L-AC-5	-171,46000*	5,95709	<.001
Young_Modulus	1L-AC-1	1L-AC-2	-3,19800*	,38520	<.001
		1L-AC-3	-,26000	,38520	,983
		1L-AC-4	-1,67400*	,38520	,003
		1L-AC-5	-7,64200*	,38520	<.001
		1L-CG-1	-2,44200*	,38520	<.001
	1L-AC-2	1L-AC-1	3,19800*	,38520	<.001
		1L-AC-3	2,93800*	,38520	<.001

Multiple Comparisons

Tukey HSD

Dependent Variable	(I) Series_code	(J) Series_code	95% Confidence Interval Lower Bound	95% Confidence Interval Upper Bound
Tensile_test	1L-AC-1	1L-AC-2	-136,3829	-99,5451
		1L-AC-3	-37,2629	-,4251
		1L-AC-4	-75,5029	-38,6651
		1L-AC-5	-288,6029	-251,7651
		1L-CG-1	-117,1429	-80,3051
	1L-AC-2	1L-AC-1	99,5451	136,3829
		1L-AC-3	80,7011	117,5389
		1L-AC-4	42,4611	79,2989
		1L-AC-5	-170,6389	-133,8011
		1L-CG-1	,8211	37,6589
	1L-AC-3	1L-AC-1	,4251	37,2629
		1L-AC-2	-117,5389	-80,7011
		1L-AC-4	-56,6589	-19,8211
		1L-AC-5	-269,7589	-232,9211
		1L-CG-1	-98,2989	-61,4611
	1L-AC-4	1L-AC-1	38,6651	75,5029
		1L-AC-2	-79,2989	-42,4611
		1L-AC-3	19,8211	56,6589
		1L-AC-5	-231,5189	-194,6811
		1L-CG-1	-60,0589	-23,2211
	1L-AC-5	1L-AC-1	251,7651	288,6029
		1L-AC-2	133,8011	170,6389
		1L-AC-3	232,9211	269,7589
		1L-AC-4	194,6811	231,5189
		1L-CG-1	153,0411	189,8789
	1L-CG-1	1L-AC-1	80,3051	117,1429
		1L-AC-2	-37,6589	-,8211
		1L-AC-3	61,4611	98,2989
		1L-AC-4	23,2211	60,0589
		1L-AC-5	-189,8789	-153,0411
Young_Modulus	1L-AC-1	1L-AC-2	-4,3890	-2,0070
		1L-AC-3	-1,4510	,9310
		1L-AC-4	-2,8650	-,4830
		1L-AC-5	-8,8330	-6,4510
		1L-CG-1	-3,6330	-1,2510
	1L-AC-2	1L-AC-1	2,0070	4,3890
		1L-AC-3	1,7470	4,1290

Multiple Comparisons

Tukey HSD

Dependent Variable	(I) Series_code	(J) Series_code	Mean Difference (I-J)	Std. Error	Sig.
		1L-AC-4	1,52400*	,38520	,007
		1L-AC-5	-4,44400*	,38520	<.001
		1L-CG-1	,75600	,38520	,391
	1L-AC-3	1L-AC-1	,26000	,38520	,983
		1L-AC-2	-2,93800*	,38520	<.001
		1L-AC-4	-1,41400*	,38520	,014
		1L-AC-5	-7,38200*	,38520	<.001
		1L-CG-1	-2,18200*	,38520	<.001
	1L-AC-4	1L-AC-1	1,67400*	,38520	,003
		1L-AC-2	-1,52400*	,38520	,007
		1L-AC-3	1,41400*	,38520	,014
		1L-AC-5	-5,96800*	,38520	<.001
		1L-CG-1	-,76800	,38520	,375
	1L-AC-5	1L-AC-1	7,64200*	,38520	<.001
		1L-AC-2	4,44400*	,38520	<.001
		1L-AC-3	7,38200*	,38520	<.001
		1L-AC-4	5,96800*	,38520	<.001
		1L-CG-1	5,20000*	,38520	<.001
	1L-CG-1	1L-AC-1	2,44200*	,38520	<.001
		1L-AC-2	-,75600	,38520	,391
		1L-AC-3	2,18200*	,38520	<.001
		1L-AC-4	,76800	,38520	,375
		1L-AC-5	-5,20000*	,38520	<.001
Flexural_strength_test	1L-AC-1	1L-AC-2	-21,08200*	4,06010	<.001
		1L-AC-3	-42,45000*	4,06010	<.001
		1L-AC-4	-20,88400*	4,06010	<.001
		1L-AC-5	-40,74800*	4,06010	<.001
		1L-CG-1	-59,33800*	4,06010	<.001
	1L-AC-2	1L-AC-1	21,08200*	4,06010	<.001
		1L-AC-3	-21,36800*	4,06010	<.001
		1L-AC-4	,19800	4,06010	1,000
		1L-AC-5	-19,66600*	4,06010	<.001
		1L-CG-1	-38,25600*	4,06010	<.001
	1L-AC-3	1L-AC-1	42,45000*	4,06010	<.001
		1L-AC-2	21,36800*	4,06010	<.001
		1L-AC-4	21,56600*	4,06010	<.001
		1L-AC-5	1,70200	4,06010	,998
		1L-CG-1	-16,88800*	4,06010	,004

Multiple Comparisons

Tukey HSD

Dependent Variable	(I) Series_code	(J) Series_code	95% Confidence Interval Lower Bound	Upper Bound
		1L-AC-4	,3330	2,7150
		1L-AC-5	-5,6350	-3,2530
		1L-CG-1	-,4350	1,9470
	1L-AC-3	1L-AC-1	-,9310	1,4510
		1L-AC-2	-4,1290	-1,7470
		1L-AC-4	-2,6050	-,2230
		1L-AC-5	-8,5730	-6,1910
		1L-CG-1	-3,3730	-,9910
	1L-AC-4	1L-AC-1	,4830	2,8650
		1L-AC-2	-2,7150	-,3330
		1L-AC-3	,2230	2,6050
		1L-AC-5	-7,1590	-4,7770
		1L-CG-1	-1,9590	,4230
	1L-AC-5	1L-AC-1	6,4510	8,8330
		1L-AC-2	3,2530	5,6350
		1L-AC-3	6,1910	8,5730
		1L-AC-4	4,7770	7,1590
		1L-CG-1	4,0090	6,3910
	1L-CG-1	1L-AC-1	1,2510	3,6330
		1L-AC-2	-1,9470	,4350
		1L-AC-3	,9910	3,3730
		1L-AC-4	-,4230	1,9590
		1L-AC-5	-6,3910	-4,0090
Flexural_strength_test	1L-AC-1	1L-AC-2	-33,6355	-8,5285
		1L-AC-3	-55,0035	-29,8965
		1L-AC-4	-33,4375	-8,3305
		1L-AC-5	-53,3015	-28,1945
		1L-CG-1	-71,8915	-46,7845
	1L-AC-2	1L-AC-1	8,5285	33,6355
		1L-AC-3	-33,9215	-8,8145
		1L-AC-4	-12,3555	12,7515
		1L-AC-5	-32,2195	-7,1125
		1L-CG-1	-50,8095	-25,7025
	1L-AC-3	1L-AC-1	29,8965	55,0035
		1L-AC-2	8,8145	33,9215
		1L-AC-4	9,0125	34,1195
		1L-AC-5	-10,8515	14,2555
		1L-CG-1	-29,4415	-4,3345

Multiple Comparisons

Tukey HSD

Dependent Variable	(I) Series_code	(J) Series_code	Mean Difference (I-J)	Std. Error	Sig.
	1L-AC-4	1L-AC-1	20,88400*	4,06010	<.001
		1L-AC-2	-,19800	4,06010	1,000
		1L-AC-3	-21,56600*	4,06010	<.001
		1L-AC-5	-19,86400*	4,06010	<.001
		1L-CG-1	-38,45400*	4,06010	<.001
	1L-AC-5	1L-AC-1	40,74800*	4,06010	<.001
		1L-AC-2	19,66600*	4,06010	<.001
		1L-AC-3	-1,70200	4,06010	,998
		1L-AC-4	19,86400*	4,06010	<.001
		1L-CG-1	-18,59000*	4,06010	,002
	1L-CG-1	1L-AC-1	59,33800*	4,06010	<.001
		1L-AC-2	38,25600*	4,06010	<.001
		1L-AC-3	16,88800*	4,06010	,004
		1L-AC-4	38,45400*	4,06010	<.001
		1L-AC-5	18,59000*	4,06010	,002
Compressive_strength_test	1L-AC-1	1L-AC-2	-1,15600	1,09281	,893
		1L-AC-3	1,19800	1,09281	,878
		1L-AC-4	-27,40000*	1,09281	<.001
		1L-AC-5	-6,30000*	1,09281	<.001
		1L-CG-1	-6,54800*	1,09281	<.001
	1L-AC-2	1L-AC-1	1,15600	1,09281	,893
		1L-AC-3	2,35400	1,09281	,295
		1L-AC-4	-26,24400*	1,09281	<.001
		1L-AC-5	-5,14400*	1,09281	,001
		1L-CG-1	-5,39200*	1,09281	<.001
	1L-AC-3	1L-AC-1	-1,19800	1,09281	,878
		1L-AC-2	-2,35400	1,09281	,295
		1L-AC-4	-28,59800*	1,09281	<.001
		1L-AC-5	-7,49800*	1,09281	<.001
		1L-CG-1	-7,74600*	1,09281	<.001
	1L-AC-4	1L-AC-1	27,40000*	1,09281	<.001
		1L-AC-2	26,24400*	1,09281	<.001
		1L-AC-3	28,59800*	1,09281	<.001
		1L-AC-5	21,10000*	1,09281	<.001
		1L-CG-1	20,85200*	1,09281	<.001
	1L-AC-5	1L-AC-1	6,30000*	1,09281	<.001
		1L-AC-2	5,14400*	1,09281	,001
		1L-AC-3	7,49800*	1,09281	<.001

Multiple Comparisons

Tukey HSD

Dependent Variable	(I) Series_code	(J) Series_code	95% Confidence Interval Lower Bound	Upper Bound
	1L-AC-4	1L-AC-1	8,3305	33,4375
		1L-AC-2	-12,7515	12,3555
		1L-AC-3	-34,1195	-9,0125
		1L-AC-5	-32,4175	-7,3105
		1L-CG-1	-51,0075	-25,9005
	1L-AC-5	1L-AC-1	28,1945	53,3015
		1L-AC-2	7,1125	32,2195
		1L-AC-3	-14,2555	10,8515
		1L-AC-4	7,3105	32,4175
		1L-CG-1	-31,1435	-6,0365
	1L-CG-1	1L-AC-1	46,7845	71,8915
		1L-AC-2	25,7025	50,8095
		1L-AC-3	4,3345	29,4415
		1L-AC-4	25,9005	51,0075
		1L-AC-5	6,0365	31,1435
Compressive_strength_test	1L-AC-1	1L-AC-2	-4,5349	2,2229
		1L-AC-3	-2,1809	4,5769
		1L-AC-4	-30,7789	-24,0211
		1L-AC-5	-9,6789	-2,9211
		1L-CG-1	-9,9269	-3,1691
	1L-AC-2	1L-AC-1	-2,2229	4,5349
		1L-AC-3	-1,0249	5,7329
		1L-AC-4	-29,6229	-22,8651
		1L-AC-5	-8,5229	-1,7651
		1L-CG-1	-8,7709	-2,0131
	1L-AC-3	1L-AC-1	-4,5769	2,1809
		1L-AC-2	-5,7329	1,0249
		1L-AC-4	-31,9769	-25,2191
		1L-AC-5	-10,8769	-4,1191
		1L-CG-1	-11,1249	-4,3671
	1L-AC-4	1L-AC-1	24,0211	30,7789
		1L-AC-2	22,8651	29,6229
		1L-AC-3	25,2191	31,9769
		1L-AC-5	17,7211	24,4789
		1L-CG-1	17,4731	24,2309
	1L-AC-5	1L-AC-1	2,9211	9,6789
		1L-AC-2	1,7651	8,5229
		1L-AC-3	4,1191	10,8769

Multiple Comparisons

Tukey HSD

Dependent Variable	(I) Series_code	(J) Series_code	Mean Difference (I-J)	Std. Error	Sig.
		1L-AC-4	-21,10000*	1,09281	<.001
		1L-CG-1	-,24800	1,09281	1,000
	1L-CG-1	1L-AC-1	6,54800*	1,09281	<.001
		1L-AC-2	5,39200*	1,09281	<.001
		1L-AC-3	7,74600*	1,09281	<.001
		1L-AC-4	-20,85200*	1,09281	<.001
		1L-AC-5	,24800	1,09281	1,000
Density_test	1L-AC-1	1L-AC-2	,03000	,01225	,179
		1L-AC-3	-,13000*	,01225	<.001
		1L-AC-4	,03000	,01225	,179
		1L-AC-5	,05000*	,01225	,005
		1L-CG-1	-,07000*	,01225	<.001
	1L-AC-2	1L-AC-1	-,03000	,01225	,179
		1L-AC-3	-,16000*	,01225	<.001
		1L-AC-4	,00000	,01225	1,000
		1L-AC-5	,02000	,01225	,586
		1L-CG-1	-,10000*	,01225	<.001
	1L-AC-3	1L-AC-1	,13000*	,01225	<.001
		1L-AC-2	,16000*	,01225	<.001
		1L-AC-4	,16000*	,01225	<.001
		1L-AC-5	,18000*	,01225	<.001
		1L-CG-1	,06000*	,01225	<.001
	1L-AC-4	1L-AC-1	-,03000	,01225	,179
		1L-AC-2	,00000	,01225	1,000
		1L-AC-3	-,16000*	,01225	<.001
		1L-AC-5	,02000	,01225	,586
		1L-CG-1	-,10000*	,01225	<.001
	1L-AC-5	1L-AC-1	-,05000*	,01225	,005
		1L-AC-2	-,02000	,01225	,586
		1L-AC-3	-,18000*	,01225	<.001
		1L-AC-4	-,02000	,01225	,586
		1L-CG-1	-,12000*	,01225	<.001
	1L-CG-1	1L-AC-1	,07000*	,01225	<.001
		1L-AC-2	,10000*	,01225	<.001
		1L-AC-3	-,06000*	,01225	<.001
		1L-AC-4	,10000*	,01225	<.001
		1L-AC-5	,12000*	,01225	<.001

Multiple Comparisons

Tukey HSD

Dependent Variable	(I) Series_code	(J) Series_code	95% Confidence Interval Lower Bound	Upper Bound
		1L-AC-4	-24,4789	-17,7211
		1L-CG-1	-3,6269	3,1309
	1L-CG-1	1L-AC-1	3,1691	9,9269
		1L-AC-2	2,0131	8,7709
		1L-AC-3	4,3671	11,1249
		1L-AC-4	-24,2309	-17,4731
		1L-AC-5	-3,1309	3,6269
Density_test	1L-AC-1	1L-AC-2	-,0079	,0679
		1L-AC-3	-,1679	-,0921
		1L-AC-4	-,0079	,0679
		1L-AC-5	,0121	,0879
		1L-CG-1	-,1079	-,0321
	1L-AC-2	1L-AC-1	-,0679	,0079
		1L-AC-3	-,1979	-,1221
		1L-AC-4	-,0379	,0379
		1L-AC-5	-,0179	,0579
		1L-CG-1	-,1379	-,0621
	1L-AC-3	1L-AC-1	,0921	,1679
		1L-AC-2	,1221	,1979
		1L-AC-4	,1221	,1979
		1L-AC-5	,1421	,2179
		1L-CG-1	,0221	,0979
	1L-AC-4	1L-AC-1	-,0679	,0079
		1L-AC-2	-,0379	,0379
		1L-AC-3	-,1979	-,1221
		1L-AC-5	-,0179	,0579
		1L-CG-1	-,1379	-,0621
	1L-AC-5	1L-AC-1	-,0879	-,0121
		1L-AC-2	-,0579	,0179
		1L-AC-3	-,2179	-,1421
		1L-AC-4	-,0579	,0179
		1L-CG-1	-,1579	-,0821
	1L-CG-1	1L-AC-1	,0321	,1079
		1L-AC-2	,0621	,1379
		1L-AC-3	-,0979	-,0221
		1L-AC-4	,0621	,1379
		1L-AC-5	,0821	,1579

*. The mean difference is significant at the 0.05 level.

Homogeneous Subsets

Tensile_test

Tukey HSD[a]

Series_code	N	Subset for alpha = 0.05					
		1	2	3	4	5	6
1L-AC-1	5	60,2160					
1L-AC-3	5		79,0600				
1L-AC-4	5			117,3000			
1L-CG-1	5				158,9400		
1L-AC-2	5					178,1800	
1L-AC-5	5						330,4000
Sig.		1,000	1,000	1,000	1,000	1,000	1,000

Means for groups in homogeneous subsets are displayed.

a. Uses Harmonic Mean Sample Size = 5,000.

Young_Modulus

Tukey HSD[a]

Series_code	N	Subset for alpha = 0.05			
		1	2	3	4
1L-AC-1	5	4,2880			
1L-AC-3	5	4,5480			
1L-AC-4	5		5,9620		
1L-CG-1	5		6,7300	6,7300	
1L-AC-2	5			7,4860	
1L-AC-5	5				11,9300
Sig.		,983	,375	,391	1,000

Means for groups in homogeneous subsets are displayed.

a. Uses Harmonic Mean Sample Size = 5,000.

Flexural_strength_test

Tukey HSD[a]

Series_code	N	Subset for alpha = 0.05			
		1	2	3	4
1L-AC-1	5	37,8000			
1L-AC-4	5		58,6840		
1L-AC-2	5		58,8820		
1L-AC-5	5			78,5480	
1L-AC-3	5			80,2500	
1L-CG-1	5				97,1380
Sig.		1,000	1,000	,998	1,000

Means for groups in homogeneous subsets are displayed.

a. Uses Harmonic Mean Sample Size = 5,000.

Compressive_strength_test

Tukey HSD[a]

Series_code	N	Subset for alpha = 0.05		
		1	2	3
1L-AC-3	5	5,4140		
1L-AC-1	5	6,6120		
1L-AC-2	5	7,7680		
1L-AC-5	5		12,9120	
1L-CG-1	5		13,1600	
1L-AC-4	5			34,0120
Sig.		,295	1,000	1,000

Means for groups in homogeneous subsets are displayed.

a. Uses Harmonic Mean Sample Size = 5,000.

Density_test

Tukey HSD[a]

Series_code	N	Subset for alpha = 0.05			
		1	2	3	4
1L-AC-5	5	1,2500			
1L-AC-2	5	1,2700	1,2700		
1L-AC-4	5	1,2700	1,2700		
1L-AC-1	5		1,3000		
1L-CG-1	5			1,3700	
1L-AC-3	5				1,4300
Sig.		,586	,179	1,000	1,000

Means for groups in homogeneous subsets are displayed.

a. Uses Harmonic Mean Sample Size = 5,000.

Means Plots

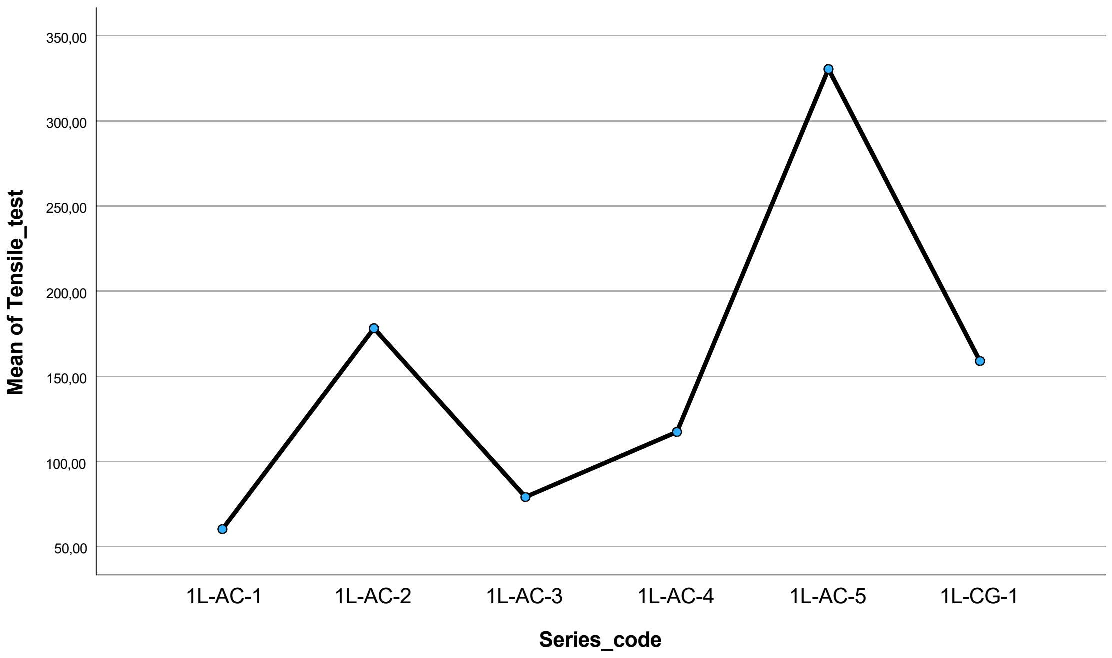
350,00
300,00
250,00
200,00
150,00
100,00
50,00
Mean of Tensile_test
1L-AC-1
1L-AC-2
1L-AC-3
1L-AC-4
1L-AC-5
1L-CG-1
Series_code

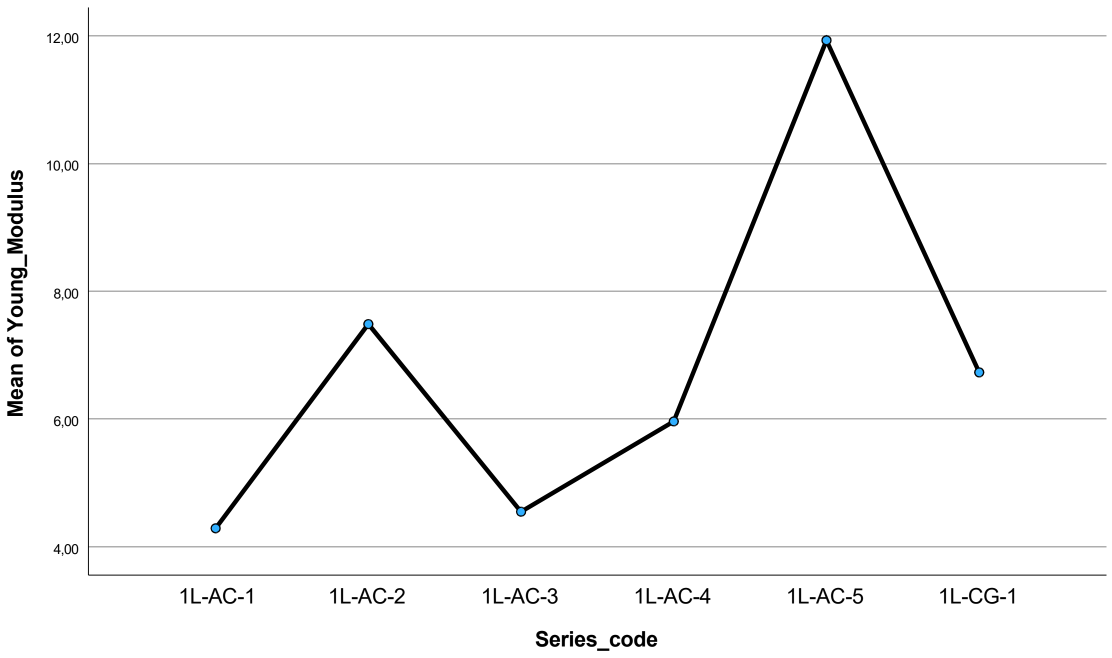
12,00
10,00
8,00
6,00
4,00
Mean of Young_Modulus
1L-AC-1
1L-AC-2
1L-AC-3
1L-AC-4
1L-AC-5
1L-CG-1
Series_code

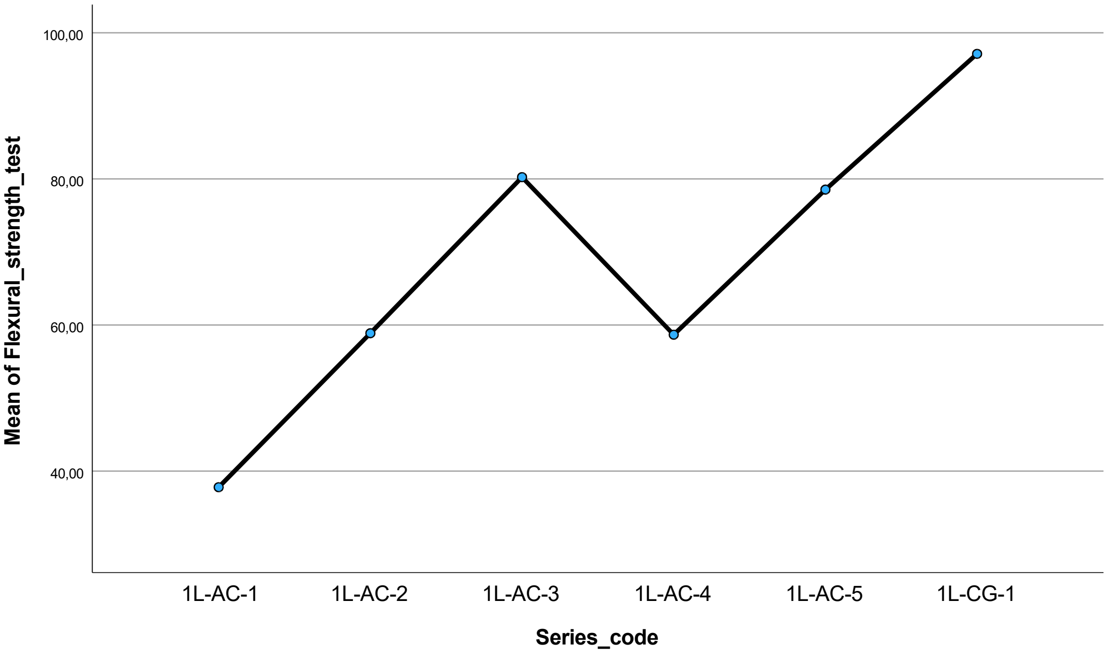

100,00
80,00
60,00
40,00
Mean of Flexural_strength_test
1L-AC-1
1L-AC-2
1L-AC-3
1L-AC-4
1L-AC-5
1L-CG-1
Series_code

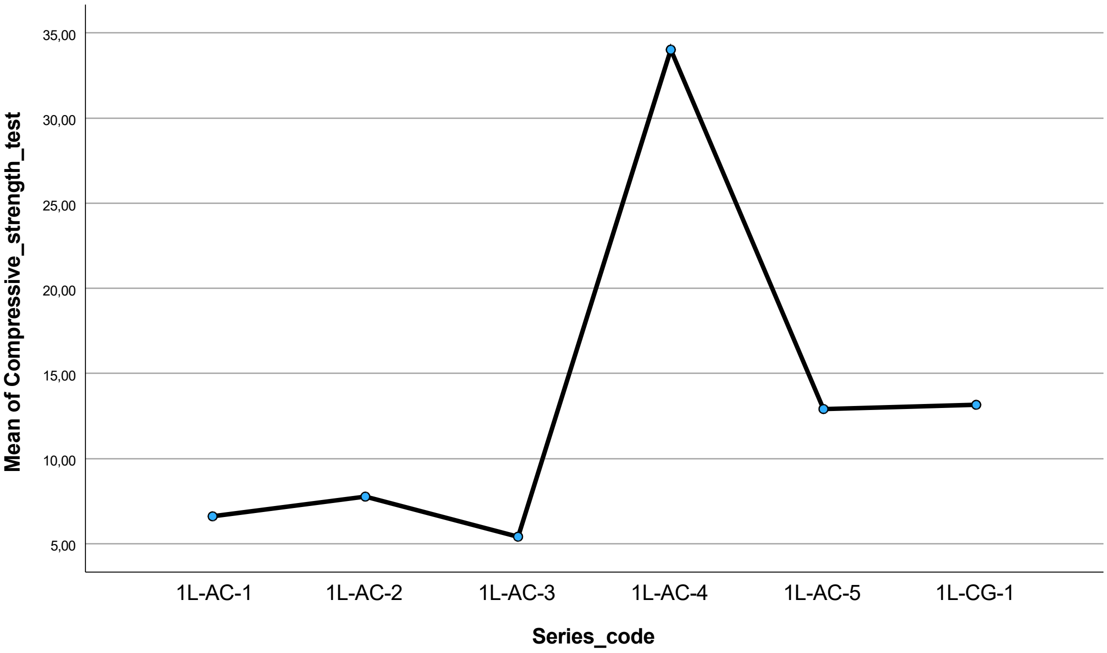

35,00
30,00
25,00
20,00
15,00
10,00
5,00
Mean of Compressive_strength_test
1L-AC-1
1L-AC-2
1L-AC-3
1L-AC-4
1L-AC-5
1L-CG-1
Series_code

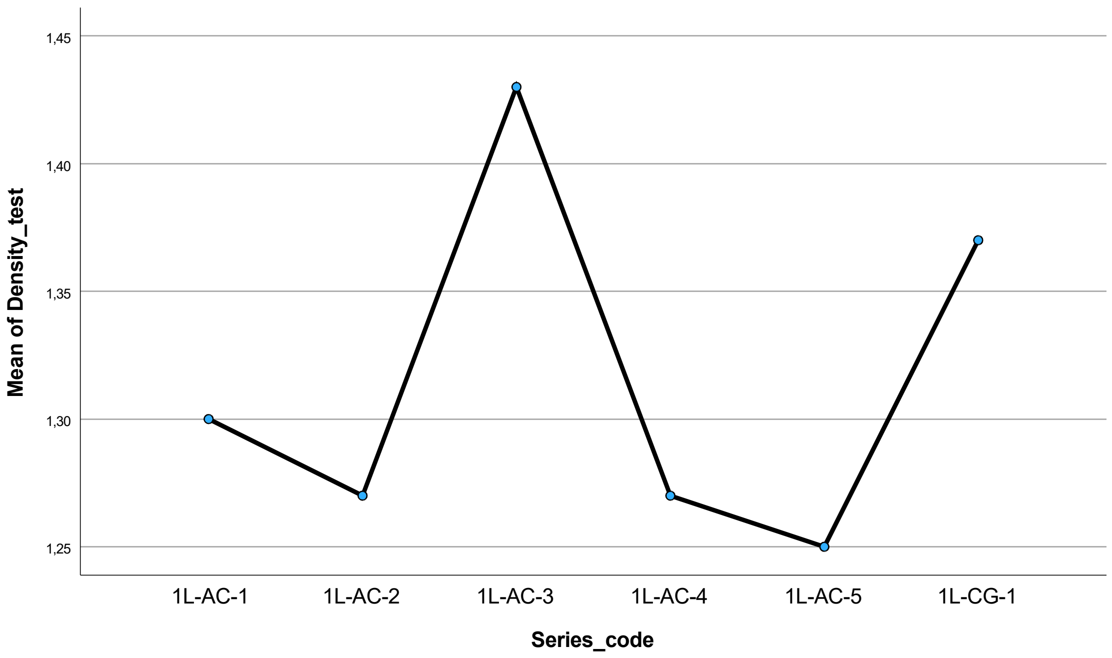
1,45
1,40
1,35
1,30
1,25
Mean of Density_test
1L-AC-1
1L-AC-2
1L-AC-3
1L-AC-4
1L-AC-5
1L-CG-1
Series_code
